# A Comparative Review of Veterinary and Human Vaccine Development Strategies: Insights into Herpesvirus Vaccinology from Latency to Elimination

**DOI:** 10.3390/vaccines14030249

**Published:** 2026-03-07

**Authors:** Guangyi Liu, Xiaoyang Zhao, Yuezhi Lin, Xiaojun Wang, Diqiu Liu

**Affiliations:** State Key Laboratory of Animal Disease Control and Prevention, Harbin Veterinary Research Institute, Chinese Academy of Agricultural Sciences, Harbin 150069, China; liuguangyi135@gmail.com (G.L.); zhaoxiaoyang2602@gmail.com (X.Z.); linyuezhi@caas.cn (Y.L.)

**Keywords:** alphaherpesvirus, vaccinology, One Health, technical bottleneck, development strategies, human and veterinary medicine

## Abstract

**Background:** Members of the virus family *Herpesviridae* are among the most successful pathogen groups in evolutionary history. They not only pose a serious public health threat to humans but also cause significant economic losses in the global livestock industry. The primary immunological challenge in developing sterilizing vaccines is the lifelong latency of herpesviruses in the nervous system or lymphoid tissues. **Methods:** This analysis compares the vaccine strategies designed against the five most important *Alphaherpesvirinae* pathogens: HSV-1/2, PRV, BHV-1, EHV-1/4, and FHV-1. The contrast between the globally licensed veterinary vaccines and the relative stagnation in the field of human HSV vaccines is stark. However, there are notable success stories regarding the implementation of ‘Marker Vaccines’ (DIVA strategies) in veterinary medicine. This review examines various vaccine modalities, assessing their potential to mitigate clinical outbreaks and their shortcomings in preventing viral shedding and establishing latency. **Results:** This study reveals common technical bottlenecks across species, attributed to immune evasion mechanisms such as the downregulation of MHC I, TAP inhibition, the failure to induce robust mucosal IgA, and safety concerns regarding the recombination of live vectors. **Conclusions:** This review highlights several promising avenues that could lead to enhanced herpesvirus vaccines and recommends the rational design of T-cell epitopes alongside innovative mucosal adjuvants. Furthermore, it bridges the gap between veterinary and human vaccinology from a One Health perspective, suggesting that lessons learned from veterinary practices could facilitate necessary breakthroughs in human medicine.

## 1. Introduction

The *Herpesviridae* family represents a ubiquitous group of large, enveloped, double-stranded DNA viruses that infect a wide spectrum of hosts, from humans to livestock [1,2]. Among these, the subfamily *Alphaherpesvirinae* is characterized by a rapid reproductive cycle, efficient destruction of infected cells, and the capacity to establish lifelong latent infections in sensory ganglia [2,3]. This unique life cycle underpins both the profound public health significance and the immense economic burden attributed to herpesvirus infections globally. The systematic review was conducted across professional databases to analyze herpesvirus vaccinology (2000–2026), targeting these pathogens as well as key technologies. Inclusion criteria focused on peer-reviewed original research, high-impact reviews, and studies emphasizing structural vaccinology or modern delivery platforms. Exclusion criteria filtered out non-primary data, English-only constraints, and outdated methodologies, except for seminal breakthroughs. This rigorous approach ensures a balanced synthesis of historical milestones and cutting-edge advancements.

The *Herpesviridae* family is systematically classified into three subfamilies based on tropism, genome organization, and replication characteristics (Table 1).

In the context of livestock agriculture, herpesviruses are agents of economic devastation. PRV, the causative agent of Aujeszky’s disease (Pseudorabies), has historically resulted in billions of dollars in losses to the global swine industry due to reproductive failure in sows and high mortality rates in piglets. Similarly, BHV-1 is a primary component of the Bovine Respiratory Disease Complex (BRDC) and is responsible for Infectious Bovine Rhinotracheitis (IBR) [4], leading to trade barriers and production losses. In equids, EHV-1/4 remains a leading cause of respiratory disorders, nervous disorders and abortion, while FHV-1 is the primary cause of viral rhinotracheitis in cats. Concomitantly, the economic impact of herpesviruses on livestock and companion animals is devastating, driving significant financial losses within the veterinary sector.

Swine: PRV (also named Suid Herpesvirus 1, SuHV-1) causes Aujeszky’s disease [5], characterized by high mortality in young piglets, reproductive failure in sows, and neurological signs at all ages [6]. Historically, PRV has necessitated massive, expensive eradication campaigns based on strict vaccination and culling programs [7,8].

Bovine: BHV-1 is the primary causative agent of IBR and Infectious Pustular Vulvovaginitis (IPV) [9,10]. IBR, often exacerbated by bacterial co-infection, is a major component of the ‘Bovine Respiratory Disease Complex’ (BRDC, or ‘shipping fever’) [11], which costs the cattle industry billions of dollars annually due to loss of appetite, reduced weight gain, and death.

Equine: EHV-1 and EHV-4 are responsible for high fever, respiratory disease, abortion storms (EHV-1), and the potentially fatal neurological syndrome [12], equine herpes myeloencephalopathy (EHM) [13,14,15]. The unpredictability of EHV-1 outbreaks poses a constant threat to breeding farms and the performance horse industry [14].

Feline: FHV-1 is the leading cause of Feline Viral Rhinotracheitis (FVR) [16,17,18], a severe component of the Feline Respiratory Disease Complex (FRDC) [19]. While rarely fatal in adults, it establishes chronic carrier states and necessitates costly, lifelong management of recurrent ocular and upper respiratory disease in a large percentage of the domestic cat population.

The veterinary viruses discussed in this review (PRV, BHV-1, EHV-1/4, FHV-1), as well as the primary human targets, HSV-1, HSV-2, and VZV, are all classified within the *Alphaherpesvirinae* subfamily. This genetic proximity implies conserved mechanisms of immune evasion and latency establishment [14,20], making comparative analysis highly informative [1]. Concurrently, HSV-1 and HSV-2 impact a significant portion of the global population, causing lifelong morbidity and increasing the risk of HIV acquisition [21,22,23]. Despite years of research, the development of a herpesvirus vaccine capable of inducing sterilizing immunity and preventing latency has remained an elusive goal. However, veterinary medicine has seen significant advancements, such as the Differentiating Infected from Vaccinated Animals (DIVA) strategy [5,24,25]. In human medicine, HSV-1 and HSV-2 are known to cause recurrent mucocutaneous lesions [26,27], while VZV is responsible for chickenpox and shingles [28,29]. Beyond these common ailments, certain human herpesviruses (HHVs) serve as potent oncogenic agents (e.g., EBV; Kaposi’s sarcoma-associated Herpesvirus, KSHV) [30,31], or are significant contributors to morbidity in immunocompromised patients, CMV [32]. Despite decades of intensive research, the development of a sterilizing prophylactic vaccine against major HHVs, particularly HSV-2, remains one of the most elusive challenges in vaccinology, underscoring fundamental gaps in our understanding of immune correlates of protection.

The substantial economic burden of these veterinary diseases, coupled with the profound clinical burden of human herpesvirus infections, necessitates a continuous and comparative reassessment of vaccine strategies. The scope of this review extends beyond species-specific literature reviews and synthesizes common challenges and breakthroughs across the *Alphaherpesvirinae* subfamily. Human herpesviruses, such as HSV and VZV, provide context as human trials often lead the way for novel vaccine platforms (e.g., mRNA technology; structural vaccinology) [33,34,35,36,37,38]. Veterinary vaccinology provides important lessons for human medicine; for instance, in certain regions, PRV, an agent of swine fever, was nearly eradicated using targeted gene-deleted vaccines. This success demonstrates that strong protective immunity can be achieved under certain conditions, highlighting a crucial field whose lessons are translatable to human health.

This review adopts a ‘One Health’ perspective to compare vaccine development across these five major pathogens (Table S1). By analyzing both the successes and failures in veterinary models, we aim to provide critical insights into the construction methods, defects, bottlenecks, and future directions of herpesvirus vaccinology. The ‘One Health’ synergy provides a strategic framework for overcoming the impasse in HSV vaccinology by drawing on historical successes in PRV control. Despite the physiological differences between hosts, alphaherpesviruses employ remarkably homologous molecular pathways for neuroinvasion and immune evasion. Key insights from PRV that are relevant to HSV translation include strategies for targeting viral spread, the development of marker vaccines, and ensuring genomic stability. Deleting the gE/gI complex—crucial for anterograde axonal transport but not for replication—effectively confines the virus to primary cells. While current HSV subunit vaccines primarily focus on entry mechanisms (such as gD), targeting gE/gI could inhibit the virus from reaching sensory ganglia. The use of negative markers (e.g., gE-deleted mRNA) allows clinicians to differentiate vaccine-induced immunity from wild-type infections, which is essential for post-market surveillance and for assessing community viral load. To mimic the stability of the Bartha-K61 PRV strain, it is necessary to advance beyond point mutations and implement large, rationally designed deletions of immune-evasion (ICP47) and spread-essential (UL37) genes to ensure safety and prevent reversion.

## 2. Biological Characteristics and Pathogenesis

The two forms of infection, lytic replication and latency with subsequent reactivation [39,40], are the reason for the complexity of herpesvirus pathogenesis.

### 2.1. The Alphaherpesvirus Lifecycle

All five viruses replicate in a similar manner [41,42,43,44]. Infection typically initiates at the mucosal epithelium, which can be located in the nasal, oral, or genital regions [45]. Viral glycoproteins facilitate the attachment and fusion with the host cells. Following lytic replication, retrograde axonal transport takes the nucleocapsid to the cell bodies, where the viral genome circularizes and persists as a non-integrated episome. A primary, productive infection establishes in mucosal or epithelial cells upon first exposure to a virus. The viral gene expression, which occurs after circularization of the linear DNA genome, occurs in a sequence of regulation: immediate-early (IE), early (E) and late (L) genes [46,47,48]. The quick disintegration of cells and the generation of many virus offspring result in substantial shedding of the virus, accompanied by severe clinical signs such as rhinitis due to BHV-1, and abortion due to EHV-1. The acute phase is characterized by the activation of neutralizing antibodies and innate immunity, which work to eliminate most of the virus.

### 2.2. Comparative Virology

A typical alphaherpesvirus virion comprises four structural components: the Core, which contains the linear, double-stranded DNA genome; the Capsid, which is an icosahedral protein structure that surrounds the core; the Tegument, an amorphous layer of regulatory and structural proteins situated between the capsid and the envelope. The key tegument proteins (e.g., VP16, VP22) play essential roles in transcription and egress [49,50,51,52] and Envelope (a lipid bilayer studded with at least 10–15 different viral glycoproteins: gB, gC, gD, gH/gL complex, etc.) [1,53,54,55,56,57,58,59].

The glycoproteins serve as the primary targets of neutralizing antibodies and cytotoxic T lymphocytes (CTLs), making them a focal point for subunit and vector-based vaccine design. Notably, the glycoprotein D (gD) is a highly conserved and essential molecule involved in host cell entry [60]. It mediates entry through interaction with cellular receptors (nectin-1 for HSV and PRV) and fusion [61,62,63]. The strong conservation of molecules such as gD and gB [64,65], the principal fusion protein, across PRV, BHV-1, and HSV provides a biological rationale for conducting homologous trials based on vaccine designs validated in other species.

### 2.3. Comparative Pathogenicity

PRV is distinct due to its broad host range, infecting cattle, dogs, and sheep as dead-end hosts, along with its extreme neurotropism [66,67,68]. In pigs, it causes neurological signs in neonates and respiratory distress in fattening pigs. BHV-1 primarily creates immunosuppression, paving the way for secondary bacterial pneumonia (*Pasteurella multocida*). It also causes abortion storms. EHV-1 is particularly noted for its endotheliotropism [69], leading to vasculitis [70,71], which results in abortion and EHM [72]. FHV-1 causes severe ocular disease (keratitis) and upper respiratory tract infection [73,74,75], often becoming chronic in shelter environments. HSV-1 and HSV-2 are largely neurotropic and mucocutaneous [76,77], with rare but severe complications, including encephalitis and neonatal herpes [78,79,80,81,82].

### 2.4. Latency and Reactivation

Crucially, during the primary infection, the virus utilizes retrograde transport to travel along sensory neurons (e.g., trigeminal ganglia, dorsal root ganglia) to the neuronal soma, where it establishes latency [52]. In this state, many viral genes are silenced. The only detectable viral transcript is the Latency Associated Transcript (LAT), a non-coding RNA that plays a key role in maintaining latency and preventing neuronal apoptosis [83].

Latency represents a significant immunological challenge for all herpesvirus vaccines. Current vaccines, even highly effective live-attenuated vaccines (LAVs), predominantly target the lytic phase by inducing strong neutralizing antibodies and CTL responses that reduce viral load and clinical manifestations. However, these vaccines consistently fail to prevent the establishment of latency or subsequent periodic reactivation. Reactivation, triggered by physiological stressors (e.g., stress, illness, immune suppression, corticosteroid use), results in anterograde transport of the virus back down the neuron, leading to localized lytic replication and viral shedding [84,85]. This recurrent shedding is the main mechanism for disease transmission in both human and animal populations. Consequently, the objective of next-generation vaccinology is to either:

## 3. Current Herpesvirus Vaccine Landscapes

Early herpesvirus vaccines relied on techniques that decreased the virulence of the virus while preserving its immunogenicity. This approach became the foundation for the development of associated licensed products for many other herpes viruses. The clinical and economic pressure exerted by alphaherpesviruses has driven the development of numerous vaccines across different hosts. However, a common theme emerges: existing vaccines are largely successful at disease control but fundamentally fail at sterilizing immunity—the complete prevention of infection and the establishment of latency. Therefore, the effectiveness of these prophylactics is best measured by their ability to reduce clinical severity, minimize viral shedding, and control large-scale outbreaks, rather than by their capacity to achieve eradication.

### 3.1. Inactivated Vaccines (Killed Viruses)

Inactivated vaccines (IVs) are produced by cultivating the virus to a high titer and subsequently treating it with chemicals, typically formalin or beta-propiolactone (BPL) or binary ethylenimine (BEI). These agents cross-link the nucleic acid, rendering the virus non-replicative while preserving the structural integrity of the viral envelope proteins. These induce high levels of circulating neutralizing antibodies (Humoral Immunity, Th_2_-biased) but are generally poor at stimulating CTLs. These vaccines are widely used for equine herpesvirus (EHV) [86,87] and feline herpesvirus (FHV) [88,89]. In equine practice, killed vaccines are preferred for pregnant mares to mitigate the risk of abortion, though their duration of immunity (DOI) is limited, often necessitating boosters every 3 to 6 months.

IVs offer the highest safety profile, as they carry no risk of reversion to virulence or establishment of latency by the vaccine strain itself. This characteristic is particularly crucial when vaccinating susceptible populations, such as pregnant mares against EHV-1 (to prevent abortion) and FHV-1 in pregnant queens. The major drawback lies in their limited immunological breadth. Chemical treatment can often distort or destroy critical conformational epitopes on surface glycoproteins, leading to suboptimal neutralizing antibody responses. Furthermore, IVs primarily elicit humoral immune response, necessitating the use of potent adjuvants (e.g., alum, oil emulsions) and multiple doses to achieve adequate titers [90]. They are notably poor inducers of the robust CTL response, which is essential for clearing lytic infection and controlling reactivated virus within the nervous system. The lack of strong cell-mediated immunity (CMI) renders IVs a suboptimal choice in scenarios where cellular immunity is the primary correlate of protection [91], such as in controlling latent BHV-1 reactivation.

### 3.2. Live-Attenuated Vaccines (LAVs)

LAVs are derived from the serial passage of wild-type strains in non-natural hosts, low-temperature cultures, or through chemical mutagenesis. This process selects for strains with diminished replication rates and reduced virulence in the target host.

LAVs are generally superior immunogens, as they mimic a natural infection, inducing sustained antigen presentation and stimulating both mucosal IgA and systemic IgG response, alone with strong CD8 and CD4 T-cell activations. The VZV Oka strain LAV (used for chickenpox) is a premier example of successful empirical attenuation [92,93], providing durable immunity by establishing a benign form of latency. Similarly, early PRV LAVs provided rapid and comprehensive protection in swine [94]. The attenuation process is empirical, relying on phenotypic changes rather than known genetic deletions, which raises several safety concerns. Notably, there is a possibility that the LAV strain could genetically revert, even partially, to a virulent phenotype, a concern previously raised with some early EHV and BHV LAVs [95,96,97]. Additionally, certain LAV strains may still cause mild disease, or, critically, induce abortion when administered to pregnant animals, such as certain BHV-1 LAVs. While attenuated, LAVs still typically establish latency, complicating eradication efforts if the attenuated strain could reactivate and spread.

### 3.3. Modified Live Vaccines (MLVs)

Modified Live Vaccines (MLVs) are the cornerstone of livestock herpesvirus control [98]. They are attenuated by passaging in cell culture or through genetic engineering. MLVs replicate in the host, presenting antigens through the Major Histocompatibility Complex Class I (MHC I) pathway, thus stimulating a robust CTL response, which is Th1-biased and essential for clearing intracellular viruses. MLVs are particularly dominant in the pig (PRV) [99] and cattle (BHV-1) sectors [100]. They provide rapid protection, often within 3 to 5 days post-vaccination.

A notable veterinary success story is the DIVA strategy, which represents the most significant advancement in herpesvirus control, primarily applied to PRV and BHV-1 [101,102]. These vaccines feature a specific deletion in the glycoprotein E (gE) gene. Since gE is non-essential for replication but critical for virulence, the virus is effectively attenuated. By utilizing a companion diagnostic ELISA, veterinarians can distinguish between animals infected with the wild-type virus (gE positive) and vaccinated animals (gE negative) [103]. This strategy has enabled China, the USA, and much of Europe to eradicate or control PRV in commercial swine herds—a remarkable achievement that has yet to be replicated in human medicine.

### 3.4. Construction Modes Vaccines

Early MLVs, such as the Bartha-K61 strain for PRV were developed through serial passage, resulting in random mutations [104]. Modern vaccines utilize ’Rational Design‘ strategies, including Thymidine Kinase (TK) Deletion [100]. The *TK* gene is essential for viral replication in non-dividing cells, such as neurons. The deletion of *TK* prevents the virus from reactivating from latency or replicating in the central nervous system, thereby functioning as a primary safety mechanism. Additionally, Glycoprotein Deletion (gE/gI) is often performed in tandem, which disrupts anterograde neuronal transport, thereby reducing viral shedding without compromising immunogenicity.

Viral vectors are employed to deliver herpesvirus antigens. Canarypox-vectored vaccines expressing EHV-1 gB, gC, and gD have received licensing [105]. These vectors are abortive in mammals, ensuring a high safety profile while inducing cellular immunity. Furthermore, adenovirus vectors were used in experimental BHV-1 vaccines to deliver cytokines, such asIL-2 or IFN-γ, alongside viral antigens to enhance efficacy [106].

Focusing on key surface glycoproteins, these molecules are primary targets for neutralizing antibodies. Current subunit approaches often utilize these recombinant proteins combined with potent adjuvants, such as molecular patterns or oil-in-water emulsions, to mimic the immunogenicity of live viruses. Glycoproteins serve as primary targets for neutralizing antibodies and CTLs, making them central to subunit and vector-based vaccine design. The molecule gD is highly conserved and essential, making it difficult to induce mutations. Furthermore, gD mediates entry into the host cell by interacting with cellular receptors, such as nectin-1 for HSV and PRV, and facilitates fusion with the virus. Molecules like gD and gB, the major fusion protein, exhibit a high degree of conservation among PRV, BHV-1 and HSV, which provides a biological rationale for applying vaccine design principles.

## 4. Effectiveness Profiles of Licensed Prophylactics

The history of herpesvirus vaccinology illustrates a significant transition from early empirical methods that focused on whole-virus attenuation to precise, molecular engineering aimed at optimizing antigen presentation and stimulating specific immune correlates. This evolution has been crucial in addressing the inherent safety and effectiveness concerns associated with the virus’s complex life cycle. The effectiveness profiles of veterinary herpesvirus vaccines are presented in Table 2.

### 4.1. PRV: The Gold Standard in Control?

PRV causes Aujeszky’s disease and, uniquely among the veterinary herpesviruses discussed herein, has been the focus of highly successful national eradication programs in countries such as the United States, Canada, and parts of Europe. This success can be directly attributed to the implementation of the DIVA strategy [5,101].

The cornerstone of PRV control is the LAV derived from the Bartha strain, which is often further modified by the deletion of non-essential glycoproteins, particularly gE and in some cases, gI. The gE-deleted LAVs retain their immunogenicity while losing the ability to express gE, a non-essential glycoprotein implicated in neurovirulence and efficient viral spread [107,108]. Vaccination induces robust humoral immunity and CMI against other key antigens (gB, gD) [109]. Crucially, these vaccines significantly reduce the severity of clinical signs in pigs and dramatically limit both the magnitude and duration of viral shedding, thereby interrupting the transmission chain.

An animal vaccinated with a gE-deleted vaccine remains uninfected with a wild-type virus and will test negative for gE antibodies using a diagnostic test officially recognized in field settings [110,111,112]. In contrast, a wild-type virus that is gE-positive will yield a positive result. This distinction enables veterinary and regulatory authorities to accurately identify genuine PRV-infected animals within a vaccinated population, facilitating the export of such infected animals and the eventual PRV-free certification for entire regions.

Although gE-LAVs effectively inhibit infection, they do not prevent the establishment of latency [113]. Latent PRV can be reactivated by stressors (e.g., transport, farrowing), leading to limited shedding and the potential for transmission. However, the reduced shedding significantly minimizes the risk of field transmission compared to wild-type infections. The Bartha strain itself has been shown to establish latency.

### 4.2. BHV-1: Controlling the Respiratory Disease Complex

BHV-1 infection is a global concern due to its association with IBR, reproductive failure, and the complex etiology of BRDC. The primary objective of BHV-1 vaccination is to mitigate disease severity and control the viral shedding associated with stress-induced reactivation, particularly under the high-stress conditions of transport, commonly referred to as shipping fever [114,115].

The BHV-1 vaccine market for BHV-1 is diversified, offering inactivated vaccines, LAVs, and gE-deleted marker vaccines. The inactivated vaccines are regarded as safe and non-reverting, and they are frequently combined with other BRDC pathogens, such as BVDV in multi-valent formulations [116]. However, these vaccines typically necessitate multiple doses to establish protective titers and primarily induce a humoral immune response (IgG) [117], which tends to wane relatively quickly, thereby providing limited protection against reactivation.

LAVs are highly immunogenic, generating superior CMI and inducing rapid protection. They are commonly administered via two routes: intramuscular (IM) injection and intranasal (IN) application [118,119,120,121]. IM LAVs provide excellent systemic immunity but carry a risk of mild disease or abortion when administered to pregnant cows, which has been a significant drawback. IN LAVs are designed to elicit strong mucosal IgA at the site of entry, offering a faster onset of protection. Their attenuation limits systemic spread, increasing safety for pregnant animals in some formulations; however, though this remains controversial across different strains.

Like PRV, BHV-1 gE-deleted vaccines are marker vaccines, providing the DIVA capability (gE) [90,122]. They are essential for herds participating in international trade or eradication efforts, as they allow for the differentiation of vaccinated healthy animals from wild-type infected animals that pose potential transmission risks.

BHV-1 vaccines have demonstrated effectiveness in the field by significantly reducing both the incidence and severity of acute IBR symptoms [123,124]. Furthermore, BHV-1 vaccination markedly decreases the quantity of virus shed following reactivation when compared to unvaccinated, latently infected animals. This reduction is crucial for controlling outbreaks, particularly in crowded feedlots or during transport. Like PRV, BHV-1 vaccines do not prevent the establishment of latency in the trigeminal ganglia. Consequently, an animal may appear clinically healthy and seropositive while still harboring the latent virus, which can reactivate due to corticosteroid treatment or stress, resulting in a transient shedding phase. Therefore, the primary goal is to mitigate shedding rather than eliminate viral carriage.

### 4.3. EHV-1 and EHV-4: The Abortigenic and Neurological Challenge

EHV-1 and EHV-4 pose dual threats: respiratory disease (EHV-4) and the more severe consequences of EHV-1, including abortion storms and EHM [125]. The current repertoire of EHV vaccines is widely criticized by equine practitioners for its perceived failure to provide robust and reliable protection against the most devastating manifestations, particularly EHM.

The commercially available EHV vaccines are almost exclusively inactivated or attenuated preparations targeting both EHV-1 and EHV-4, often formulated with powerful adjuvants (Montanide™, Quil-A, QS-21, ISCOMs, CpG ODN, MPL, Flagellin). The most common regimen used in pregnant mares follows a rigorous schedule (typically during the 5th, 7th, and 9th months of gestation) to prevent EHV-1-induced abortion [126]. These vaccines induce high neutralizing antibody titers [127,128]. Less common, but available in some markets, are highly immunogenic vaccines that carry the theoretical risk of shedding and reversion, which necessitates caution in their use.

EHV-1 vaccines provide adequate protection against the mild respiratory form of the disease and are considered moderately effective at reducing the risk of abortion [129]. However, they have two significant shortcomings: the inability to prevent infection and latency, and the failure to combat EHM [125]. Vaccinated horses can still become infected with EHV-1 and establish latency. The primary function of the vaccine is to enhance systemic antibodies that may intercept the virus before it spreads to the placenta or the central nervous system (CNS). A major limitation is the inability of current vaccines to protect against the paralytic form, EHM, which is caused by the neuropathogenic strain of EHV-1 that harbors a single-nucleotide polymorphism (SNP) resulting in the D752 form of the DNA polymerase [130]. The pathogenesis of EHM is associated with significant vasculitis in the CNS, often necessitating high levels of cell-mediated immunity to contain. Current vaccines generally do not induce robust CD8^+^ T-cell responses, which are essential for rapidly clearing the infection within the endothelium or ganglia [131]. The consensus is that EHV vaccines serve as a risk management tool, reducing herd transmission and the incidence of abortion, but they do not confer the sterilizing immunity necessary to protect individuals against neurovirulent strains [132].

### 4.4. FHV-1: Management of the Carrier State

FHV-1 is responsible for FVR, which significantly contributes to the population of chronically infected carrier cats. Vaccination serves as a crucial strategy in multi-cat environments, such as shelters and catteries. FHV-1 vaccines are typically included in tri-valent or quad-valent core vaccines, commonly referred to as FVRCP (Feline Viral Rhinotracheitis, Calicivirus, Panleukopenia) [133,134]. Both inactivated and LAVs are available, with administration predominantly occurring subcutaneously [135].

LAVs provide the most rapid and comprehensive protection against clinical signs. They are highly immunogenic but cannot be used in pregnant queens. In contrast, inactivated vaccines are safer for pregnant animals but are less immunogenic, often requiring strong adjuvants and frequently failing to prevent infection or shedding entirely. FHV-1 vaccines are considered highly effective in reducing the severity of clinical disease, such as ocular discharge, sneezing, and conjunctivitis, associated with primary infection and subsequent reactivation [98,136,137].

In vaccinated cats, the duration of viral shedding and the severity of clinical signs are significantly shorter and milder, respectively, compared to unvaccinated cohorts. Importantly, no commercial FHV-1 vaccine prevents infection or the establishment of lifelong latency in the trigeminal ganglia. Many vaccinated cats can still become latent carriers. Furthermore, the immunity induced is relatively short-lived, necessitating yearly or even semi-annual boosters to maintain protective titers against the lytic cycle.

### 4.5. Human Herpesviruses: The Divergence in Success

The field of human vaccinology offers a contrasting narrative, showcasing both a significant success story with the VZV and a protracted costly failure with the HSV. The application of LAV technology in the VZV pneumonia vaccine has greatly benefited public health. The Oka strain live-attenuated varicella (chickenpox) vaccine is highly effective in preventing primary infections, has drastically reduced the incidence of childhood chickenpox, and mitigates the long-term complications associated with shingles.

Zoster (Shingles) vaccines include Zostavax (LAV) and Shingrix (subunit, adjuvanted) [138]. Zostavax is a highly concentrated version of the Oka strain, specifically designed to enhance immune response in the elderly and prevent reactivation of the virus (shingles). However, its effectiveness diminishes over time. In contrast, Shingrix is a non-live, recombinant subunit vaccine that combines the VZV glycoprotein E (gE) with a robust adjuvant system (AS01B) [139]. Shingrix elicits a strong and durable CD4^+^ T-cell response, providing over 90% protection against shingles for more than seven years. This success is attributed to the combination of a high-quality antigen (gE) and a potent adjuvant that fosters a robust CMI necessary for controlling the latent virus. In stark contrast, the HSV-2 vaccine exemplifies a persistent failure; despite being the first virus targeted for a subunit vaccine over four decades ago, no prophylactic vaccine against HSV-2 is currently licensed.

Most clinical candidates, such as GSK’s gD-AS04, have focused on the essential gD to prevent genital disease caused by HSV-2. However, Phase III trials have consistently demonstrated that these vaccines do not provide protection against either infection or disease in the target population, specifically, HSV-negative women [140,141]. While some candidates exhibited transient effectiveness in a subset of individuals, the overall protection remained inadequate. This indicates that gD alone is an insufficient antigen, as the resulting immunity, which is primarily humoral, fails to address the critical issue of HSV’s rapid spread and neuroinvasion. The failure of these candidates underscores the necessity for sterilizing immunity at the mucosal surface to achieve protective immunity against HSV-a goal none of the subunit candidates could achieve.

## 5. Critical Analysis of Existing Limitations

The ‘Holy Grail’ of vaccinology is sterilizing immunity which completely blocks the virus from entering cells. Current herpesvirus vaccines are considered ‘leaky’; they prevent clinical disease (morbidity) but do not prevent infection. A vaccinated animal challenged with the corresponding herpesvirus will still replicate the virus in the nasal mucosa and shed it to herd mates, thereby maintaining the chain of transmission. These limitations arise from the molecular interplay of the virus, the virus’s ability to hide, and the failure of the immune system’s failure to mount a defense at the anatomical sites where sterilizing immunity is required.

### 5.1. The Latency Paradox and the Failure of Sterilizing Immunity

The most significant shortcoming of current herpesvirus vaccines is their universal failure to prevent the establishment of a lifelong latent state [142,143]. To prevent latency, the immune response must achieve sterilizing immunity at the mucosal surface that is powerful enough to neutralize or clear the entire initial viral inoculum before it can penetrate the basement membrane and reach the peripheral nerve endings [144,145]. Inactivated and many subunit vaccines primarily induce systemic neutralizing IgG. However, this IgG is often too slow or present in insufficient concentration at the mucosal surface to intercept the high-titer initial inoculum [146,147]. Furthermore, the short, intense burst of replication during primary infection—especially in aggressive pathogens like HSV-2 and EHV-1—ensures that the virus utilizes retrograde transport to the ganglia before systemic immunity peaks [148,149].

While LAVs induce robust CD8^+^ T cell responses, even these take several days to mature and migrate to the site of infection. This delay is problematic; studies confirm that HSV-1 can reach the ganglia of the trigeminal nerve within hours of inoculation. The establishment of latency in sensory neurons (trigeminal, sacral, etc.) creates an anatomical and immunological sanctuary [150]. Neurons typically exhibit low MHC I expression and are largely protected from direct immune-mediated killing, particularly by circulating CTLs [151].

MHC I downregulation in neurons represents a core immune evasion strategy employed by herpesviruses. Healthy mature neurons inherently exhibit low baseline MHC I expression that is region- and subtype-specific, primarily modulating synaptic plasticity and neural circuit homeostasis rather than canonical antigen presentation. This low expression forms the biological foundation for further pathogen-mediated MHC I suppression, allowing evasion of CD8^+^ T cell recognition. However, neuronal MHC I can be robustly induced by IFN-γ through the JAK-STAT1-IRF1 signaling axis. Neurotropic viruses antagonize this pathway at multiple transcriptional and post-translational levels, effectively blocking MHC I upregulation even in high IFN-γ inflammatory microenvironments, thereby sustaining long-term immune escape. Notably, a critical research gap remains unaddressed: the role of NK cells in responding to neuronal MHC I downregulation, particularly regarding the “missing self” response, NK cell activation thresholds, and their dual roles in viral control versus off-target neuronal damage, which are systematically overlooked in the current literature. Furthermore, while CD4^+^ T cells help in peripheral ganglia is phenotypically associated with controlling latent neurotropic infections, the underlying mechanisms—including cytokine-mediated restoration of MHC I expression, support for glial antigen presentation, and non-cytolytic antiviral functions—remain largely uncharacterized, severely limiting the development of targeted neuro-immunotherapies and vaccines.

Herpesviruses establish latency in the trigeminal ganglia. While vaccination significantly reduces the viral load during reactivation and subsequent shedding, the vaccine-induced CTLs are incapable of eliminating the reservoir of infected neurons during the latent phase. The failure to maintain a sufficient density and quality of these CD8^+^ T cells is the reason herpesvirus latency is never cleared, necessitating continuous surveillance in cattle herds [152,153,154]. The complete failure of any vaccine candidate to prevent latency in human trials underscores the complexity of the issue [141,155]. Initial infections are often subclinical; however, the virus still successfully invades the sacral ganglia, indicating that the host’s natural immune responses and those enhanced by vaccines at the genital mucosa are functionally inadequate against the viral entry mechanism.

### 5.2. Duration of Immunity (DOI) and the Need for Chronic Boosting

A key measure of vaccine quality is the longevity of the protective immune response. For many herpesvirus vaccines, particularly those used in veterinary medicine, the DOI is disappointingly short, necessitating frequent and costly booster vaccinations [86,131,156,157,158]. Inactivated vaccines (IVs), lacking the sustained antigen presentation characteristic of replicating viruses, often generate a transient humoral response reliant on short-lived plasma cells due to poor memory induction. This brief DOI highlights the challenges associated with inducing robust, long-lived memory B cells and T cells in the absence of replicating antigens. The dependence on transient antibodies renders the animal susceptible to breakthrough infections, especially during periods of stress when EHV-1 or FHV-1 may reactivate. In contrast, the VZV-LAV (Oka strain) provides protection for decades, largely due to its ability to establish a benign latency that offers periodic, low-level endogenous boosting of the immune system.

The necessity for frequent boosting imposes a significant economic and logistical burden on the livestock industry. In large commercial beef and dairy operations (BHV-1) and extensive swine farming (PRV control), the costs associated with frequent handling, injections, and labor for boosting can undermine compliance and overall profitability. Consequently, the short DOI effectively transforms the vaccine from a preventative measure into a chronic management expense.

### 5.3. The ‘Leaky’ Vaccine Phenomenon and Viral Evolution

A ‘leaky’ vaccine is one that reduces clinical signs in the vaccinated host but fails to prevent infection or transmission, allowing the pathogen to replicate and shed at subclinical levels. This characteristic problem is common among many herpesvirus prophylactics [159].

The goal of PRV and BHV-1 marker vaccines is to significantly reduce the magnitude and duration of viral shedding upon reactivation [109,160,161,162]. While these vaccines achieve this goal, the shedding is not eliminated. In densely populated environments, such as feedlots or large swine confinement buildings, even reduced shedding from a large percentage of the vaccinated population can sustain the virus within the herd. This subclinical transmission is challenging to detect and complicates eradication efforts, demonstrating that a reduction in disease severity is not synonymous with an effective transmission block.

The EHV-1 inactivated vaccine is considered ‘leaky’ against infection [148,163,164,165]. Although the vaccine mitigates the acute respiratory disease but fails to contain systemic spread, leaving the most devastating complication unresolved [129,166]. While controversial, it has been hypothesized that the widespread use of vaccines primarily targeting highly cell-associated neuropathogenic (D752) EHV-1 strains capable of causing EHM, as the vaccine immunity does not effectively contain the systemic phase of this variant [167]. This phenomenon highlights the danger of relying on vaccines that only provide partial protection against multi-phenotypic viruses [126,167,168]. A major theoretical and practical concern regarding leaky vaccines is the potential for them to impose selection pressure on the wild-type virus, thereby driving the evolution of vaccine-escape mutants. In regions where the Bartha-K61 LAV has been used extensively [169,170], field reports have documented the emergence of PRV variants with altered virulence or transmission characteristics, potentially selected by the widespread, yet incomplete, immune pressure [171].

### 5.4. Safety, Recombination, and Adverse Event Concerns

Historically, some temperature-sensitive mutants have reverted to virulence. Evidence suggests that live attenuated vaccine strains can recombine with wild-type strains in the field [172], potentially creating novel, virulent genotypes. Some MLVs for PRV and BHV-1 may cause transient immunosuppression, making the herd susceptible to secondary bacterial infections (BHV-1-*Mannheimia haemolytica*, *Pasteurella multocida*, and *Histophilus somni*; PRV- *Streptococcus suis*). While these vaccines are generally safe, licensed herpesvirus vaccines are not without inherent risks, which must be carefully weighed against their benefits, especially in high-value animals or immunocompromised human populations.

The biggest safety concern associated with LAVs (e.g., VZV Oka, certain BHV-1 and FHV-1 attenuated strains) is the potential for reversion to virulence and homologous recombination [173,174]. The co-circulation of a LAV and a latent wild-type virus within the same cell theoretically creates an opportunity for genetic exchange through homologous recombination, which could lead to the emergence of novel, highly virulent strains that possess replication advantages of the LAV alongside the virulence determinants of the wild-type strain. This risk is acknowledged in the development of new PRV and BHV-1 LAVs and is typically mitigated by removing multiple virulence genes.

The successful VZV subunit vaccine (Shingrix) is quite reactive and causes pain, redness and fever which is short lived. The acceptable threshold of reactogenicity is particularly low for the HSV subunit candidate, where more potent adjuvants needed to drive a protective CMI response are not useful [175]. FHV-1 and EHV-1 vaccines contain aluminum or oil-based adjuvants that are well-known for causing severe local reactions, as well as adverse effects in cats [176,177]. These adjuvants have been implicated in the development of feline injection-site sarcomas (FISS), a fatal tumor [178]. This risk has set off a major re-evaluation of adjuvant use in feline vaccinology, often favoring safer but less effective alternatives.

These undesirable effects are often expected and are generally considered an indicator of the vaccine’s “take” or potency, which include pain, heat, and swelling at the injection site and transient systemic effects comprising lethargy, mild fever, and reduced feed intake for 24–48 h post-vaccination as well as decreased productivity that means the temporary drop in milk yield in dairy cows or a slight reduction in growth rates in swine. However, some adverse reactions are “unacceptable” risks that often lead to batch recalls or market withdrawal, including anaphylaxis, vaccine-induced abortion, and reversion to virulence as well as FISS.

## 6. Examining and Decoding Bottlenecks in Designing Herpesvirus Vaccine

The major technical and biological challenges hinder the existing disease-controlling vaccines from achieving sterilizing immunity. The herpesviruses encounter barriers due to their evolved tactics to evade the immune system of the host. Furthermore, these viruses are difficult to measure and to induce the required immune responses.

### 6.1. Viral Blinding of Host Defenses

Alphaherpesviruses possess a distinctive set of genes specifically designed to obstruct, commandeer, or restrict the hosts innate and adaptive immune responses. For a next-generation vaccine to be effective, it must address these evasion strategies at the molecular level. CTLs require the presentation of viral peptides on the surface of affected cells by MHC I molecules. Herpesviruses use numerous mechanisms to bypass this crucial pathway, causing the infection of any CTL cell.

HSV-1 and HSV-2 produce the immediate early protein ICP47, a potent and highly selective inhibitor of the Transporter Associated with Antigen Processing (TAP) [179,180]. ICP47 competitively binds to TAP, thereby preventing the translocation of viral peptides from the cytosol into the endoplasmic reticulum (ER) for loading onto MHC I molecules. The homologous viruses PRV (pUL49.5) and BHV-1 (gM/pUL49.5) employ different but functionally similar mechanisms. The UL49.5 protein complex also inhibits TAP transport and obstructs the exit of MHC I from the ER [181,182]. Herpesviruses encode homologs of cellular anti-apoptotic proteins, such as Bcl-2, to prolong the survival of infected cells, allowing them to function as viral factories [183]. The glycoprotein G (gG) of BHV-1 and gG of PRV exhibit a similar reactivity, potentially due to their interaction with pro-inflammatory chemokines like IL-8 and RANTES [184,185]. As a result, the virus gains an advantage in spreading and invading the nervous system, as immune cells—such as T cells, neutrophils and dendritic cells, which are not effectively recruited to the site of primary infection due to these chemical signals.

Additionally, herpesviruses disrupt the innate immune response by interfering with the complement system and directly diminishing the neutralizing capacity of antibodies. The gC protein of HSV [186,187], BHV-1 [188], and EHV-1 [189,190] act as a viral decoy receptor that binds to complement components like C3b, inhibiting complement-mediated lysis and opsonization. Consequently, a vaccine must elicit adequate neutralizing antibody (NAb) titers to counteract this systemic complement evasion mechanism, a challenge that few existing inactivated or subunit vaccines consistently meet.

### 6.2. Mucosal Barrier

The primary route of infection for all five herpesviruses under review is mucosal (respiratory, ocular, or genital). Therefore, achieving sterilizing immunity fundamentally requires a robust and maintained immune response at the mucosal surface. The challenge in attaining this represents a significant bottleneck. Most vaccines are administered intramuscularly (IM), inducing systemic IgG; however, herpesviruses primarily target mucosal surfaces. This results in a ‘compartmentalization’ bottleneck where systemic IgG does not transudate effectively to the nasal or vaginal mucosa [191,192,193]. Vaccines should be administered directly to the respiratory or nasal mucosa (e.g., certain BHV-1 LAVs), targeting the Mucosa-Associated Lymphoid Tissue (MALT) to stimulate B cells to switch to IgA production. The challenge lies in ensuring sufficient antigen uptake without causing local inflammatory side effects. Tissue-resident memory T (T_RM_) cells are CD8^+^ T cells that establish permanent residence in the epithelial and sub-epithelial layers of barrier tissues (skin, mucosa). Current systemic vaccines are relatively inefficient at generating long-lasting, high-density T_RM_ populations at the specific anatomical site of viral entry and reactivation [194,195].

EHV-1 and FHV-1 primarily cause respiratory disease, yet their vaccines often fail to provide durable protection against nasal shedding. This suggests a failure to induce long-term IgA responses. The rapid turnover of epithelial cells and the constant environmental exposure in livestock and equine settings necessitate an extremely robust and continually renewed T_RM_ pool, which current attenuated or inactivated formulations do not effectively provide.

### 6.3. Defining CoP

The development of new vaccines is severely hampered by the lack of clearly defined, universally accepted, and biologically validated Correlates of Protection (CoP). Without a reliable CoP, effectiveness testing becomes expensive, slow, and reliant on costly Phase III trials. For many years, vaccine development was guided by the principle that high NAb titers equate to protection. However, the repeated failure of HSV-2 subunit vaccines, which generated high NAb levels but failed in Phase III trials, has demonstrated that NAb alone is an insufficient CoP for herpesviruses, particularly those that establish latency. The success of VZV Shingrix clearly illustrated that the CoP for controlling reactivation of latent infection is not NAb titer, but the frequency and functionality of antigen-specific CD4^+^ T cells. CD4^+^ T cells are critical for maintaining the CD8^+^ T cell surveillance in the ganglia. The primary goal of the vaccine is to maintain T-cell surveillance to prevent or abort reactivation [28].

The true CoP for many alphaherpesviruses likely resides in the functional quality and anatomical location of T cells. The bottleneck is the development of standardized, high-throughput assays that can accurately measure the ability of a single T cell to simultaneously produce multiple cytokines (IFN-γ, IL-2, TNF-α) or perform cytotoxic killing. Additionally, assays must identify T cells with the specific markers for residence (CD103+, CD69+) at mucosal and ganglionic sites [196,197]. For multiplex vaccines, it is crucial to confirm that the T cell response is broad enough to recognize potential viral escape mutants.

The CoP characterization should be considered as prioritized aspects. Future studies should focus on defining quantitative thresholds for protective T_RM_ cell density is foundational, as it establishes standardized benchmarks for the minimal cellular abundance needed to block pathogen challenge at entry sites. Additionally, the metrics of functional polyfunctionality metrics, assessed via intracellular cytokine staining (ICS) assays and tetramer staining, provide robust, biologically validated readouts of antigen-specific immune activity directly linked to in vivo protective effectiveness. Furthermore, the systematic vaccinology approaches enable the development of multivalent and multicomponent vaccines, overcoming the limitation of traditional single-marker assessments. It is also critical to account for innate immune imprinting is critical, as prior immune exposures shape baseline and vaccine-induced responses, thus altering the generalizability of CoP across diverse populations. Together, these advances will establish a well-defined CoP to accelerate next-generation vaccine development.

### 6.4. Animal Model Limitations and Translational Hurdles

Bridging the gap between proof-of-concept in the laboratory and successful field deployment is complicated by the inadequacy of available animal models for specific herpesvirus pathologies. While small animal models are convenient, they often fail to replicate the natural history of the infection. The EHV-1 model in mice or hamsters cannot fully replicate the key pathological events in horses, such as the EHM vasculitis and thrombosis that occur in the spinal cord. Consequently, vaccine candidates must be extensively tested in the natural host (horses), a process that is extremely expensive, logistically complex, and subject to high variability, which severely slows research and development for new EHM-specific prophylactics.

While human trials face immense ethical scrutiny, veterinary vaccinology must navigate the high costs associated with large animal trials (e.g., cattle and swine) and strict regulatory requirements, particularly concerning the stability of DIVA marker genes in the field. The necessity of demonstrating effectiveness against field challenge in the natural host remains the most significant translational bottleneck for novel BHV-1 and PRV candidates.

## 7. Future Development Directions

The limitations outlined in the preceding sections demonstrate that incremental improvements to classical vaccine paradigms will not suffice to achieve sterilizing immunity against alphaherpesviruses. The future of herpesvirus vaccinology must pivot towards rational, structure-based design and advanced delivery systems that specifically address immune evasion, mucosal defense, and the control of the latent reservoir. This strategy necessitates the integration of breakthroughs from human virology, such as mRNA technology, with the high-throughput challenges associated with veterinary deployment. The development of vaccines and therapeutic interventions for herpes simplex virus (HSV) infections, specifically GEN-003 and HSV529, marks a significant advancement in mitigating the global burden of HSV-related diseases. GEN-003, a therapeutic vaccine, and HSV529, a prophylactic vaccine, have been central to clinical trials designed to decrease both the frequency and severity of HSV outbreaks.

### 7.1. Structural Vaccinology and Rational Antigen Design

Future success hinges on moving beyond the use of full-length viral proteins as antigens and instead focusing on stabilizing the specific conformational epitopes that induce the most potent neutralizing antibodies and T cell responses [84,198,199].

The critical event in the herpesvirus lifecycle is the fusion of the viral envelope with the host cell membrane, mediated by a highly conserved complex of glycoproteins: gB (the core fusion protein) and the gH/gL dimer. For many enveloped viruses, such as RSV and influenza, stabilizing the metastable pre-fusion conformation of the fusion protein has yielded substantially more potent vaccines than the post-fusion state. BHV-1 and PRV gB are prime candidates for this approach. By engineering these proteins to be conformationally ‘locked’ in the pre-fusion state—the form recognized before the irreversible structural change upon membrane binding—vaccines can elicit higher titers of broadly neutralizing antibodies that block viral entry more effectively. This technique promises to overcome the ‘low-affinity’ antibody problem common to many current subunit vaccines.

gD remains essential for entry and is highly immunogenic; however, past HSV trials demonstrated that its full-length form was insufficient. Rational design focuses on generating truncated, epitope-focused gD variants that are optimally presented for B-cell activation. Using high-resolution structural biology (cryo-EM, X-ray crystallography), researchers can now pinpoint conserved neutralizing epitopes across species. For example, designing a gD protein that forces the presentation of the key receptor-binding loop could be applied to HSV, PRV, and BHV-1 to elicit superior neutralizing antibodies, potentially overcoming the low mucosal antibody titers that currently plague protection.

### 7.2. The T-Cell Mission: Targeting T_RM_ and Ganglia Surveillance

Due to the inability to halt latency, the revised objective is no longer primary prevention but rather the control of reactivation and the immediate elimination of infected cells. Functional T-cell surveillance must be established at critical anatomical sites [200]. Upcoming T-cell epitope-based vaccines should advance beyond neutralizing antibodies by identifying T_RM_-stimulating epitopes through rational design. The development of T-cell epitope-based vaccines represents a promising frontier in immunotherapy, particularly in overcoming the limitations of neutralizing antibodies. These vaccines utilize specific immunogenic peptides to elicit robust T-cell responses, providing enhanced effectiveness and safety compared to traditional vaccines. Techniques that deposit T_RM_ cells in the vaginal or nasal mucosa and sensory ganglia may serve as the first line of defense, effectively targeting the virus as soon as it enters, potentially preventing latency. The natural latency control mechanism of the human VZV immunity can be referred to as the ‘Pull and Keep’ strategy for T_RM_.

The delivery route is crucial; employing appropriate adjuvants (such as TLR agonists-CpG ODN, MPL, Flagellin) alongside intranasal or epicutaneous/transdermal delivery this is likely to preferentially induce the formation of T_RM_ cells expressing CD103 and CD69 markers. A vaccine for EHV-1 or BHV-1, administered intranasally with a heat-labile adjuvant, could aim to inundate the nasal cavities with T_RM_ for immediate high-density cellular surveillance, capable of neutralizing or clearing the virus before it spreads to the trigeminal ganglia. The successful development of the VZV Shingrix vaccine underscores the essential roles of functional CD4^+^ T-cells in maintaining the quality and longevity of the CD8^+^ T-cell response in the ganglia. For long-term safety surveillance, a variety of T-cell epitopes (not solely the primary neutralizing antigen) and adjuvants (Quil-A, QS-21, ISCOMs) that strongly promote Th1 polarization (via IFNγ production) are included.

### 7.3. Advanced Delivery Systems: Nanoparticles and VLPs

The effectiveness of any rationally designed antigen depends critically on its successful delivery and presentation to the immune system. Advanced nanotechnologies significantly enhance both safety and immunogenicity. The utilization of nanoparticles, such as chitosan and PLGA [201], for antigen encapsulation facilitates intranasal delivery by protecting the antigen from degradation and promoting uptake by mucosa-associated lymphoid tissue (MALT). This strategy represents a promising approach to obstructing viral entry at the initial point of contact.

Virus-like particles (VLPs) are self-assembling nanostructures that replicate the native viral architecture while lacking the genetic material necessary for replication, thus offering a high safety profile. They inherently present multiple antigens in their native, highly repetitive orientation, maximizing the cross-linking of B-cell and T-cell receptors. VLPs can be engineered to display various critical herpesvirus antigens, including gD, gB, and gH, simultaneously on their surface. This approach is particularly promising for complex pathogens such as EHV-1 and FHV-1, which may necessitate immune responses against multiple targets to effectively prevent both respiratory and systemic disease manifestations. Currently, VLP-based BHV-1 candidates are under investigation to provide superior CMI without the associated risks of live attenuated vaccines (LAVs).

The lipid nanoparticle (LNP) encapsulation system for mRNA vaccines has revolutionized the field of vaccinology by enabling safe and highly efficient delivery into antigen-presenting cells (APCs) [202]. The LNP, along with the intracellular synthesis of the antigen, functions as an in situ adjuvant, eliciting a robust innate immune response essential for CTL priming [203]. This technology is rapidly being deployed for HSV-2 and VZV candidates, delivering speed and purity that surpass traditional culture methods.

### 7.4. The Versatility of Nucleic Acid Multiplexing

The limitations of single-antigen subunit vaccines can be effectively addressed by leveraging the versatility of mRNA and DNA platforms. Unlike traditional vaccines that rely solely on a single gD antigen, mRNA vaccines facilitate the simultaneous co-expression of multiple strategically selected antigens, including gD, gB, gH/gL, and potentially a T-cell-focused tegument protein. This innovative approach enables the customization of the immune response to target various disease outcomes. For BHV-1, one construct could emphasize antigens that block respiratory entry (gD) while another component targets proteins necessary for neuronal spread (gE), thereby minimizing latency establishment. For EHV-1, a multiplex vaccine could target both the entry mechanism (gD) and the vascular spread mechanism, addressing the EHM threat. The rapid manufacturing capabilities of nucleic acid vaccines present a significant advantage for veterinary medicine, especially in the face of sudden, high-morbidity outbreaks such as EHV-1abortion storms. The ability to swiftly design and deploy an mRNA vaccine targeting a specific outbreak strain functions as a powerful tool for controlling epizootics.

### 7.5. Therapeutic Vaccines: A Shift in Strategy

Given the biological challenges associated with preventing initial infection and latency, the most achievable goal for the immediate future may be the development of therapeutic vaccines [204]. By focusing on the latently infected host, therapeutic vaccines are administered to animals or humans who are already latently infected, with the aim of enhancing T-cell surveillance and reducing both the frequency and severity of symptomatic reactivation and viral shedding [205]. The development of DNA vaccines for therapeutic use against herpes simplex virus (HSV) has garnered significant attention due to their potential to provide both prophylactic and therapeutic benefits. A notable example is the Vaxfectin^®^-adjuvanted HSV-2 plasmid DNA vaccine, which has demonstrated effectiveness in both prophylactic and therapeutic contexts in animal models.

The therapeutic mRNA vaccine could specifically target the minimization of painful genital lesions and, critically, reduce the subclinical viral shedding that drives transmission. This approach would effectively disrupt the chain of infection within the human population without requiring sterilizing immunity. Furthermore, a highly effective T-cell boosting vaccine could diminish the recurrence rate and severity of FVR in carrier cats, thereby transforming chronic management into proactive prevention. This therapeutic strategy employs immunological tools to address the latency paradox, shifting the focus from the challenging objective of eradicating the virus to the more attainable objectives of achieving a functional cure and controlling transmission.

The feasibility of DNA vaccines and T-cell epitope-based vaccines in reducing viral shedding compared to lesion frequency in veterinary populations represents a promising research avenue, especially considering the limitations of traditional vaccines in managing certain infectious diseases. The advancement of T-cell epitope-based vaccines marks a significant leap in vaccine technology, providing a targeted approach that utilizes specific immunogenic peptides to elicit strong T-cell responses. This strategy not only improves vaccine effectiveness but also reduces the likelihood of adverse side effects, a crucial consideration in veterinary applications where animal welfare is of utmost importance.

### 7.6. Synthesis of the Current Landscape and the Dichotomy of Success

The persistent global prevalence and significant economic burden imposed by alphaherpesviruses—from HSVs in humans to PRV, BHV-1, EHV-1/4, and FHV-1 in livestock—underscore a central, unresolved dilemma in modern vaccinology. This comprehensive review meticulously details the evolution of vaccine construction paradigms, transitioning from empirical attenuation to precision genetic engineering. However, the core challenge remains unchanged: no licensed prophylactic vaccine can induce sterilizing immunity or prevent the establishment of latency. So far, the journey has yielded tools for disease mitigation rather than eradication.

The analysis of existing herpesvirus vaccines reveals a functional dichotomy in their effectiveness. In terms of epidemiological control, the success of the PRV eradication program in many developed nations represents a monumental achievement in veterinary vaccinology. The gene-deleted LAV, combined with the DIVA strategy, has provided the necessary tools to manage viral spread and certify herd health. Achieving herd immunity against alphaherpesviruses (e.g., PRV, BHV-1) requires near-100% vaccination coverage due to high transmissibility in intensive farming. Unlike sterilizing immunity, current veterinary vaccines are often leaky, focusing on reducing clinical shedding and environmental viral load rather than preventing infection. Success hinges on a “transmission-blocking” effect to suppress epidemics.

Beyond biology, veterinary herd immunity is structural; strategies like “All-In/All-Out” (AIAO) systems synchronize population immunity to prevent the introduction of naive hosts. Furthermore, managing Colostrum-Derived Immunity (CDI) is critical. While maternal antibodies provide early protection, they often interfere with neonatal vaccination—a bottleneck addressed by next-generation intranasal vaccines. Crucially, because latency is never cleared, herd immunity remains immunologically fragile. Ceasing vaccination triggers catastrophic “rebound” outbreaks from the latent reservoir, necessitating a permanent commitment to cycle-based vigilance in eradication programs.

The functional non-sterilizing vaccine can achieve near-eradication when coupled with robust regulatory frameworks and testing. Conversely, focusing on immunological control of latency, the success of the VZV subunit vaccine, Shingrix, marks a breakthrough in the treatment of latent infections. Shingrix offers compelling evidence that a rationally designed, adjuvanted subunit vaccine can effectively enhance CD4 T-cell surveillance, thereby controlling the reactivation of a latent virus and significantly preventing disease recurrence (shingles).

However, these two successes stand in stark contrast to the failures observed in the field. The chronic re-emergence of EHV-1 abortion storms, the limited DOI provided by FHV-1 vaccines, and the repeated Phase III failures of HSV-2 candidates all stem from similar molecular bottlenecks: immune evasion through MHC I downregulation, inadequate induction of mucosal T cell response induction, and reliance on inadequate neutralizing antibodies as the sole CoP.

## 8. Conclusions

The complexity of host–pathogen co-evolution is evident in the ongoing battle against herpesviruses. Despite veterinary medicine having pioneered the successful deployment of the DIVA/Marker vaccines for PRV and BHV-1, sterilizing immunity remains unachieved across all species. The path forward for the ‘One Health’ approach is synergistic. Overcoming the technical obstacles of mucosal delivery and immune evasion using mRNA platforms and rational epitope design, next-generation vaccines may finally be able to do more than control disease, but stop establishment of the silent threat latency.

In conclusion, we must look beyond the empirical quest for a perfect attenuated strain to envision the future of herpesvirus vaccinology. This future calls for an advanced strategy, driven by molecular considerations, that recognizes the relentless nature of latency as a biological constant and focuses on functional cure: controlling the virus through superior T-cell surveillance to eliminate disease severity and abolish transmission. Through rational antigen design, advanced adjuvants and nucleic acid technology the next decade is poised to witness the development of effective transmission-blocking and therapeutic vaccines that can finally master the ubiquitous alphaherpesvirus.

## Figures and Tables

**Table 1:** The *Herpesviridae* family and representative viruses.

Subfamily	Defining Characteristic	Representative Viruses
*Alphaherpesvirinae*	Fast replication cycle; lytic infection in epithelial cells; latency in sensory ganglia.	HSV-1/2 (herpes simplex virus 1 and herpes simplex virus 2), VZV (varicella zoster virus), PRV (pseudorabies virus), BHV-1 (bovine herpesvirus type 1), EHV-1/4 (equine herpesvirus 1 and equine herpesvirus 4), FHV-1 (feline herpesvirus type 1)
*Betaherpesvirinae*	Slow replication cycle; latency in secretory glands, lymphoreticular cells.	CMV (cytomegalovirus)
*Gammaherpesvirinae*	Lymphoid tropism; latency in B or T cells.	EBV (Epstein–Barr virus)

**Table 2:** The current features of licensed veterinary herpesvirus vaccines.

Virus	Primary Vaccine Type	Effectiveness Measure	Latency Prevention?	Key Limitation
PRV	Live-attenuated (gE deleted)	Disease control, reduced shedding, DIVA.	No	Requires strict herd management, latency persists.
BHV-1	Live-attenuated (gE deleted)	Reduced IBR severity, reduced shedding upon reactivation.	No	Inactivated forms require frequent boosting; LAVs risk abortion.
EHV-1/4	Inactivated	Reduces abortion risk and respiratory signs.	No	Poor protection against EHM/neuropathogenic strains.
FHV-1	LAV/Inactivated	Reduces the severity of FVR/clinical signs.	No	Does not prevent carrier state; short duration of immunity.
HSV-1/2	Subunit (gD)	None licensed.	No	Repeated clinical trial failures; humoral response insufficient for mucosal protection.

## Data Availability

The original contributions presented in this study are included in the article and Supplementary Materials. Further inquiries can be directed to the corresponding authors.

## Supplementary Materials

The following supporting information can be downloaded at: https://www.mdpi.com/article/10.3390/vaccines14030249/s1, Table S1: Comparative Frameworks in Human and Veterinary Vaccinology of Alphaherpesvirus.

## References

[1] Brito A.F., Pinney J.W. (2020). The evolution of protein domain repertoires: Shedding light on the origins of the Herpesviridae family. Virus Evol..

[2] Duan S.H., Li Z.M., Yu X.J., Li D. (2025). Alphaherpesvirus in Pets and Livestock. Microorganisms.

[3] Kuny C.V., Szpara M.L. (2021). Alphaherpesvirus Genomics: Past, Present and Future. Curr. Issues Mol. Biol..

[4] Grissett G.P., White B.J., Larson R.L. (2015). Structured literature review of responses of cattle to viral and bacterial pathogens causing bovine respiratory disease complex. J. Vet. Intern. Med..

[5] Mettenleiter T.C. (2020). Aujeszky’s Disease and the Development of the Marker/DIVA Vaccination Concept. Pathogens.

[6] Chen H., Fan J., Sun X., Xie R., Song W., Zhao Y., Yang T., Cao Y., Yu S., Wei C. (2023). Characterization of Pseudorabies Virus Associated with Severe Respiratory and Neuronal Signs in Old Pigs. Transbound. Emerg. Dis..

[7] Bo Z., Li X. (2022). A Review of Pseudorabies Virus Variants: Genomics, Vaccination, Transmission, and Zoonotic Potential. Viruses.

[8] Zheng H.H., Fu P.F., Chen H.Y., Wang Z.Y. (2022). Pseudorabies Virus: From Pathogenesis to Prevention Strategies. Viruses.

[9] Sibhat B., Ayelet G., Skjerve E., Gebremedhin E.Z., Asmare K. (2018). Bovine herpesvirus-1 in three major milk sheds of Ethiopia: Serostatus and association with reproductive disorders in dairy cattle. Prev. Vet. Med..

[10] Raaperi K., Orro T., Viltrop A. (2014). Epidemiology and control of bovine herpesvirus 1 infection in Europe. Vet. J..

[11] Chen X., Wang X., Qi Y., Wen X., Li C., Liu X., Ni H. (2018). Meta-analysis of prevalence of bovine herpes virus 1 in cattle in Mainland China. Acta Trop..

[12] Mohamed E., Zarak I., Vereecke N., Theuns S., Laval K., Nauwynck H. (2025). Genomic analysis and replication kinetics of the closely related EHV-1 neuropathogenic 21P40 and abortigenic 97P70 strains. Vet. Res..

[13] Stokol T., Soboll Hussey G. (2019). Editorial: Current Research in Equid Herpesvirus Type-1 (EHV-1). Front. Vet. Sci..

[14] Laval K., Poelaert K.C.K., Van Cleemput J., Zhao J., Vandekerckhove A.P., Gryspeerdt A.C., Garre B., van der Meulen K., Baghi H.B., Dubale H.N. (2021). The Pathogenesis and Immune Evasive Mechanisms of Equine Herpesvirus Type 1. Front. Microbiol..

[15] Duran M.C., Suazo M., Maturana A., Vargas M.P., Garcia A., Ahumada C., Pezoa A., Goehring L.S., Lara F. (2025). First Equine Herpes Myeloencephalopathy (EHM) Outbreak in Chile. Animals.

[16] Jiang Y., Lai Z., Dai L., Deng Y., Zhong L., Li S., Lu G. (2025). Protective efficacy of inactivated FHV-1 vaccine in cats following challenge with the Chinese field strains. Front. Vet. Sci..

[17] Yang M., Mu B., Ma H., Xue H., Song Y., Zhu K., Hao J., Liu D., Li W., Zhang Y. (2024). The Latest Prevalence, Isolation, and Molecular Characteristics of Feline Herpesvirus Type 1 in Yanji City, China. Vet. Sci..

[18] Cavalheiro J.B., Echeverria J.T., Ramos C.A.N., Babo-Terra V.J. (2023). Frequency of feline herpesvirus 1 (FHV-1) in domestic cats from Campo Grande, MS, Brazil. An. Acad. Bras. Cienc..

[19] Zheng H., Yue H., Wang B., Yu X., Liu Y., Yu J., Zhang J., Han K., Han Y., Su H. (2025). An efficient method for the selective isolation of feline herpesvirus 1(FHV-1) in feline calicivirus (FCV) coinfected specimens. BMC Vet. Res..

[20] Koyanagi N., Kawaguchi Y. (2020). Evasion of the Cell-Mediated Immune Response by Alphaherpesviruses. Viruses.

[21] Scrofani S., D’Avanzo P.A., Lewis K.I., Weiser-Schlesinger A., Guo T.Y., Krause K.D., Halkitis P.N. (2025). Prevalence of HSV-1 and HSV-2 among young gay and bisexual men and their association with HIV and other risk factors: Findings from the P18 Cohort Study. AIDS Care.

[22] Shi H.J., Kim-Jeon M.D., Kim J.H., Kim K.A., Park Y., Lee J., Hong S.H., Song J., Kim M.H., Kwon M. (2025). Unveiling the Hidden Burden of TORCH Infections in HIV Patients: A Residual Serum Seroepidemiological Study in a Korean Metropolitan City. Infect. Chemother..

[23] Aldunate M., Tyssen D., Johnson A., Latham C.F., Ellenberg P., Cowieson N., Hayward J.A., Center R.J., Ramsland P.A., Hearps A.C. (2025). Lactic acid produced by optimal vaginal Lactobacillus spp. potently and specifically inactivates HIV-1 in vitro by targeting the viral RNA genome and reverse transcriptase. PLoS Pathog..

[24] Alhaj M. (2016). Safety and Efficacy Profile of Commercial Veterinary Vaccines against Rift Valley Fever: A Review Study. J. Immunol. Res..

[25] Francis M.J. (2018). Recent Advances in Vaccine Technologies. Vet. Clin. N. Am. Small Anim. Pract..

[26] Hedley A., Bullard J., Van Caeseele P., Shaw S., Tsang R., Alexander D.C., Dust K., Stein D.R. (2024). A case for implementing an HSV1/2, VZV, and syphilis lesion panel in Manitoba, Canada. Microbiol. Spectr..

[27] Sam S.S., Caliendo A.M., Ingersoll J., Abdul-Ali D., Kraft C.S. (2018). Performance evaluation of the Aptima HSV-1 and 2 assay for the detection of HSV in cutaneous and mucocutaneous lesion specimens. J. Clin. Virol..

[28] Aschner C.B., Herold B.C. (2021). Alphaherpesvirus Vaccines. Curr. Issues Mol. Biol..

[29] Livieratos A., Schiro L.E., Gogos C., Ntaios G., Akinosoglou K. (2025). Varicella Zoster Virus and Stroke: An Intricate Relationship. Viruses.

[30] Chuckpaiwong V., Phimpho P., Lekhanont K., Kaewkorn P., Jongkhajornpong P. (2024). Epstein-Barr Virus Keratouveitis-Induced Malignant Glaucoma After Penetrating Keratoplasty: A Case Report and Literature Review. Ocul. Immunol. Inflamm..

[31] Mularoni A., Cona A., Mikulska M., Pecoraro F., Piazza C., De Vita E., Pietrosi G., Bulati M., Lazzarotto T., Luppi M. (2026). HHV-8/KSHV in Solid Organ Transplantation: Current Gaps of Knowledge and Future Directions. Transpl. Infect. Dis..

[32] Ahmed A., Al Saiyan Z., Subarna R.T., Naser N.M., Khan N., Bhattacharjee B., Deen N.S. (2026). Prevalence of CMV, EBV, HPV, and HSV among South Asian healthy population: A systematic review and meta-analysis. PLoS Glob. Public Health.

[33] Lukac D.M., Yuan Y., Arvin A., Campadelli-Fiume G., Mocarski E., Moore P.S., Roizman B., Whitley R., Yamanishi K. (2007). Reactivation and lytic replication of KSHV. Human Herpesviruses: Biology, Therapy, and Immunoprophylaxis.

[34] Abdelgawad A., Damiani A., Ho S.Y., Strauss G., Szentiks C.A., East M.L., Osterrieder N., Greenwood A.D. (2016). Zebra Alphaherpesviruses (EHV-1 and EHV-9): Genetic Diversity, Latency and Co-Infections. Viruses.

[35] Mettenleiter T.C., Klupp B.G., Bamford D.H., Zuckerman M. (2021). Pseudorabies Virus (Herpesviridae). Encyclopedia of Virology.

[36] Soboll Hussey G., Osterrieder N., Azab W., Bamford D.H., Zuckerman M. (2021). Equine Herpesviruses (Herpesviridae). Encyclopedia of Virology.

[37] Studdert M.J., Granoff A., Webster R.G. (1999). Bovine Herpesvirus (Herpesviridae). Encyclopedia of Virology.

[38] Sykes J.E., Lappin M.R., Thomasy S.M., Beatty J.A., Sykes J.E. (2021). 34—Feline Herpesvirus Infections. Greene’s Infectious Diseases of the Dog and Cat.

[39] Hay J., Ruyechan W.T., Arvin A., Campadelli-Fiume G., Mocarski E., Moore P.S., Roizman B., Whitley R., Yamanishi K. (2007). Alphaherpesvirus DNA replication. Human Herpesviruses: Biology, Therapy, and Immunoprophylaxis.

[40] Tombacz D., Maroti Z., Olah P., Dormo A., Gulyas G., Kalmar T., Csabai Z., Boldogkoi Z. (2025). Temporal transcriptional profiling of host cells infected by a veterinary alphaherpesvirus using nanopore sequencing. Sci. Rep..

[41] Wu B.W., Engel E.A., Enquist L.W. (2014). Characterization of a replication-incompetent pseudorabies virus mutant lacking the sole immediate early gene IE180. mBio.

[42] Tombacz D., Kakuk B., Torma G., Csabai Z., Gulyas G., Tamas V., Zadori Z., Jefferson V.A., Meyer F., Boldogkoi Z. (2022). In-Depth Temporal Transcriptome Profiling of an Alphaherpesvirus Using Nanopore Sequencing. Viruses.

[43] Wu L., Cheng A., Wang M., Jia R., Yang Q., Wu Y., Zhu D., Zhao X., Chen S., Liu M. (2020). Alphaherpesvirus Major Tegument Protein VP22: Its Precise Function in the Viral Life Cycle. Front. Microbiol..

[44] Ma Z., Liu D., Cao W., Guo L., Liu K., Bai J., Li X., Jiang P., Liu X. (2024). Suppression of ZBP1-mediated NLRP3 inflammasome by the tegument protein VP22 facilitates pseudorabies virus infection. mBio.

[45] Fan D., Wang M., Cheng A., Jia R., Yang Q., Wu Y., Zhu D., Zhao X., Chen S., Liu M. (2020). The Role of VP16 in the Life Cycle of Alphaherpesviruses. Front. Microbiol..

[46] Sawtell N.M., Thompson R.L. (2021). Alphaherpesvirus Latency and Reactivation with a Focus on Herpes Simplex Virus. Curr. Issues Mol. Biol..

[47] Enquist L.W., Tomishima M.J., Gross S., Smith G.A. (2002). Directional spread of an alpha-herpesvirus in the nervous system. Vet. Microbiol..

[48] Tomishima M.J., Enquist L.W. (2002). In vivo egress of an alphaherpesvirus from axons. J. Virol..

[49] Browne H., Bell S., Minson T. (2004). Analysis of the requirement for glycoprotein m in herpes simplex virus type 1 morphogenesis. J. Virol..

[50] Liu W., Yang C., Tian J., Cheng J., Zhou L., Iqbal M., Mehboob A., Daines R., Zhang S., Chen M. (2026). A bivalent subunit bovine herpesvirus 1 vaccine based on the ectodomains of glycoproteins D and B elicits robust protective immunity against infection in a rabbit model. Vet. Q..

[51] Diwaker D., Kim D., Cordova-Martinez D., Pujari N., Jordan B.A., Smith G.A., Wilson D.W. (2025). The gE/gI complex is necessary for kinesin-1 recruitment during alphaherpesvirus egress from neurons. J. Virol..

[52] Roller R.J., Johnson D.C. (2021). Herpesvirus Nuclear Egress across the Outer Nuclear Membrane. Viruses.

[53] Vallbracht M., Lotzsch H., Klupp B.G., Fuchs W., Vollmer B., Grunewald K., Backovic M., Rey F.A., Mettenleiter T.C. (2021). In Vitro Viral Evolution Identifies a Critical Residue in the Alphaherpesvirus Fusion Glycoprotein B Ectodomain That Controls gH/gL-Independent Entry. mBio.

[54] Cwick J.P., Owen J.E., Kochetkova I., Hain K.S., Horssen N.V., Taylor M.P. (2022). Superinfection Exclusion of Alphaherpesviruses Interferes with Virion Trafficking. Microbiol. Spectr..

[55] Chen Y., Wang H., Zhou J., Liang C., Liu H., Wu S., Qi Y., Zhu X., Liu E., Wang A. (2026). The co-application of nectin-1 and PRV gD monoclonal antibodies: An effective approach to block pseudorabies virus invasion. J. Virol. Methods.

[56] Korff V., El-Debs I., Klupp B.G., Teifke J.P., Mettenleiter T.C., Sehl-Ewert J. (2025). Neurotropism of alphaherpesviruses is most prominent in the mesiotemporal, piriform and prefrontal cortices in mice. Neuroscience.

[57] Rahangdale R., Ghormode P., Tender T., Balireddy S., Birangal S., Kishore R., Mohammad F.S., Pasupuleti M., Chandrashekar H.R. (2025). Anti-HSV activity of nectin-1-derived peptides targeting HSV gD: An in-silico and in-vitro approach. J. Biomol. Struct. Dyn..

[58] Grabowska K., Wachalska M., Graul M., Rychlowski M., Bienkowska-Szewczyk K., Lipinska A.D. (2020). Alphaherpesvirus gB Homologs Are Targeted to Extracellular Vesicles, but They Differentially Affect MHC Class II Molecules. Viruses.

[59] Oliver S.L., Xing Y., Chen D.H., Roh S.H., Pintilie G.D., Bushnell D.A., Sommer M.H., Yang E., Carfi A., Chiu W. (2021). The N-terminus of varicella-zoster virus glycoprotein B has a functional role in fusion. PLoS Pathog..

[60] Xu K., Wang X.H., Ku Y.P., Guo J.Y., Fan S.H., Xue M.M., Wang J., Guo S., Pan J.J., Chu B.B. (2025). Drebrin Is Involved in the Life Cycle of Pseudorabies Virus by Regulating the Actin Cytoskeleton. Microorganisms.

[61] Chen X., Yu Z. (2024). Insight into the Interaction Mechanism of Pseudorabies Virus Infection. Biology.

[62] Duan Y., Liu N., Tan L., Gao L., Zhu G., Tao Z., Liu B., Wang A., Yao J. (2025). Isolation and biological characterization of a variant pseudorabies virus strain from goats in Yunnan Province, China. Vet. Res. Commun..

[63] Ruan L., Li L., Yang R., You A., Khan M.Z., Yu Y., Chen L., Li Y., Liu G., Wang C. (2025). Equine Herpesvirus-1 Induced Respiratory Disease in Dezhou Donkey Foals: Case Study from China, 2024. Vet. Sci..

[64] Smith K.C., Whitwell K.E., Binns M.M., Dolby C.A., Hannant D., Mumford J.A. (1992). Abortion of virologically negative foetuses following experimental challenge of pregnant pony mares with equid herpesvirus 1. Equine Vet. J..

[65] Frampton A.R., Smith P.M., Zhang Y., Grafton W.D., Matsumura T., Osterrieder N., O’Callaghan D.J. (2004). Meningoencephalitis in mice infected with an equine herpesvirus 1 strain KyA recombinant expressing glycoprotein I and glycoprotein E. Virus Genes.

[66] Wilson M.E., Holz C.L., Kopec A.K., Dau J.J., Luyendyk J.P., Soboll Hussey G. (2019). Coagulation parameters following equine herpesvirus type 1 infection in horses. Equine Vet. J..

[67] White C.N., Jones G., Baker S., Dean R.S., Brennan M.L. (2018). Variation in the Reported Management of Canine Prolapsed Nictitans Gland and Feline Herpetic Keratitis. Vet. Sci..

[68] Deng M., Liang H., Xu Y., Shi Q., Bao F., Mei C., Dai Z., Huang X. (2024). Identification, Genetic Characterization, and Pathogenicity of Three Feline Herpesvirus Type 1 Isolates from Domestic Cats in China. Vet. Sci..

[69] Gao J., Li Y., Xie Q., Al-Zaban M.I., Al-Saeed F.A., Shati A.A., Al-Doaiss A.A., Ahmed A.E., Nawaz S., Ebrahem H. (2023). Epidemiological Investigation of Feline Upper Respiratory Tract Infection Encourages a Geographically Specific FCV Vaccine. Vet. Sci..

[70] Kuang S., He Z., Zhang J., Li S., Ding J., Ma Z., Zhang B. (2025). HSV-1 hijacks mitochondrial dynamics: Potential molecular mechanisms linking viral infection to neurodegenerative disorders. Apoptosis.

[71] Marshall C., Khatoon N., Lynch E. (2021). Disseminated Herpes Simplex Virus in an Immunocompetent Patient Without Presence of Epidermal or Mucosal Lesions. Am. J. Forensic Med. Pathol..

[72] Resaz M., Parodi C., Panzeri S., Ramaglia A., Vallejos Espindola J., Severino M., Tortora D., Nobile G., Castagnola E., Mancardi M. (2026). Cortical melt sign: A novel imaging biomarker for pediatric herpes simplex encephalitis. Neuroradiology.

[73] Solomon T., Hooper C., Easton A., Rosala-Hallas A., Facer B., Moore P., Keller S.S., Whitfield T., Fernandez C., Kneen R. (2026). Safety and efficacy of adjunct dexamethasone in adults with herpes simplex virus encephalitis in the UK (DexEnceph): A multicentre, observer-blind, randomised, phase 3, controlled trial. Lancet Neurol..

[74] Mahalingam V.D., Jaworski A., Rayes O. (2025). Fatal Disseminated Neonatal Herpes Simplex Virus Type 1 Infection. Am. J. Forensic Med. Pathol..

[75] Tadros N., Godiwala N. (2025). Case Report: Disseminated herpes simplex virus complicated by hemophagocytic lymphohistiocytosis in a neonate. Front. Pediatr..

[76] Lewis K.A., Klausner J.D., Harris C.M. (2026). Neonatal Herpes Simplex Virus Infection in the United States: Regional and Time Trends, 2017–2021. Sex. Transm. Dis..

[77] El-Mayet F., Jones C. (2024). Stress Can Induce Bovine Alpha-Herpesvirus 1 (BoHV-1) Reactivation from Latency. Viruses.

[78] Ostler J.B., Jones C. (2023). The Bovine Herpesvirus 1 Latency-Reactivation Cycle, a Chronic Problem in the Cattle Industry. Viruses.

[79] Bloom D.C. (2016). Alphaherpesvirus Latency: A Dynamic State of Transcription and Reactivation. Adv. Virus Res..

[80] Cho Y., Lee C., Park S.I., Kim Y., Lee Y.S., Lee S., Yoon S., Roh G., Ha D., Oh A. (2025). Design and immunogenicity of a quadrivalent mRNA vaccine targeting HSV-2 with comparative evaluation of co-formulated and admixed formulations. Front. Immunol..

[81] Cao H., Hu J., Zeng F., Luan N., Gao D., Lei Z., Cheng J., Liu C. (2025). A Comparative Study on Immune Protection Efficacy: An HSV-1 Trivalent Antigen Subunit Vaccine Formulated with a Cellular Immunity-Inducing Adjuvant Versus an mRNA Vaccine. Vaccines.

[82] Chen Y., Liu Q., Wang C., Chen S., Li S., Liu J., Luo L., Zhang Z., Cai J., Huang M. (2026). Herpes zoster mRNA vaccine elicits superior immune responses over licensed vaccines with a favorable safety profile in animal models. Vaccine.

[83] Jang E.J., Xayaheuang S., Hwang J.Y., Kim Y., Lee K.M., Choi S.T., Kwak H.W., Nam J.H., Kim K., Yoon B. (2025). Varicella zoster virus mRNA vaccine candidate induced superior cellular immunity and comparable humoral and Fc-mediated immunity compared to the licensed subunit vaccine in a mouse model. Hum. Vaccines Immunother..

[84] Kumar R., Maji S., Tiwari S., Misra J., Gupta J., Kumar N., Gupta R., Jha N.K. (2025). Precision vaccine design targeting the prefusion state of viral glycoproteins: Advances in structural vaccinology. Biochem. Pharmacol..

[85] Samantaray M., Pushan S.S., Rajagopalan M., Abrol K., Basumatari J., Murthy T.P.K., Ramaswamy A. (2025). Designing a multi-epitope vaccine candidate against pandemic influenza a virus: An immunoinformatics and structural vaccinology approach. Mol. Divers..

[86] Warda F.F., Ahmed H.E.S., Shafik N.G., Mikhael C.A., Abd-ElAziz H.M.G., Mohammed W.A., Shosha E.A. (2021). Application of equine herpesvirus-1 vaccine inactivated by both formaldehyde and binary ethylenimine in equine. Vet. World.

[87] Attili A.R., Colognato R., Preziuso S., Moriconi M., Valentini S., Petrini S., De Mia G.M., Cuteri V. (2020). Evaluation of Three Different Vaccination Protocols against EHV1/EHV4 Infection in Mares: Double Blind, Randomized Clinical Trial. Vaccines.

[88] Wu H., Li X., Cui N., Cao Y., Liu C., Ding H., Chen Y., Yang Y., Chen X., Su X. (2025). Novel Strain-Based Triple Inactivated Vaccine Confers Rapid Neutralizing Immunity to Feline Multisystemic Pathogens With Two-Dose Regimen. Transbound. Emerg. Dis..

[89] Wang Y., Liu Y., Qiao P., Wu H., Liu C., Yang Y., Cao Y., Cui N., Wang L., Huang M. (2025). Evaluating triple inactivated vaccine-induced immunity from a large-scale study in feline population. Sci. Rep..

[90] Kerkhofs P., Renjifo X., Toussaint J.F., Letellier C., Vanopdenbosch E., Wellemans G. (2003). Enhancement of the immune response and virological protection of calves against bovine herpesvirus type 1 with an inactivated gE-deleted vaccine. Vet. Rec..

[91] El-Sheikh M.E., El-Mekawy M.F., Eisa M.I., Abouzeid N.Z., Abdelmonim M.I., Bennour E.M., Yousef S.G. (2024). Effect of two different commercial vaccines against bovine respiratory disease on cell-mediated immunity in Holstein cattle. Open Vet. J..

[92] Tommasi C., Drousioti A., Breuer J. (2025). The live attenuated varicella-zoster virus vaccine vOka: Molecular and cellular biology of its skin attenuation. Hum. Vaccines Immunother..

[93] Zhang Y., Wang S., Li G., Shi J., Chang X., Zhang H., Zhu F., Li J., Pan H., Sun J. (2025). Immunogenicity and safety of a live attenuated varicella vaccine in healthy subjects aged between 13 to 55 years: A double-blind, randomized, active-controlled phase III clinical trial in China. Expert Rev. Vaccines.

[94] Nie Z., Zhu S., Wu L., Sun R., Shu J., He Y., Feng H. (2023). Progress on innate immune evasion and live attenuated vaccine of pseudorabies virus. Front. Microbiol..

[95] Kutumbetov L., Myrzakhmetova B., Tussipova A., Zhapparova G., Tlenchiyeva T., Bissenbayeva K., Nurabayev S., Kerimbayev A. (2025). Development and Preclinical Evaluation of a Lyophilized Vaccine Against Equine Herpesvirus Type 4 (EHV-4). Vaccines.

[96] Hu Y., Zhang S.Y., Sun W.C., Feng Y.R., Gong H.R., Ran D.L., Zhang B.Z., Liu J.H. (2024). Breaking Latent Infection: How ORF37/38-Deletion Mutants Offer New Hope against EHV-1 Neuropathogenicity. Viruses.

[97] Ohtsuka H., Yamaguchi T., Maeda Y., Tomioka M., Tajima M. (2020). Effect of administering activated lymphocytes originated from the dam on the immune cell reaction in Holstein calves. Pol. J. Vet. Sci..

[98] Wu H., Qiao P., Chen Y., Liu C., Huo N., Ding H., Wang X., Wang L., Xi X., Liu Y. (2024). Cellular and humoral immune responses in cats vaccinated with feline herpesvirus 1 modified live virus vaccine. Front. Vet. Sci..

[99] Zhou M., Abid M., Cao S., Zhu S. (2023). Recombinant Pseudorabies Virus Usage in Vaccine Development against Swine Infectious Disease. Viruses.

[100] Zhang S., Liu G., Wang C., Guo A., Chen Y. (2025). Enhanced immunogenicity of a BoHV-1 gG-/tk- vaccine. Vaccine.

[101] Galleguillos-Becerra C., Cardenas M., Vasquez-Martinez Y., Tapia F., Yanez Z., Cancino T., Valdes I., Cortez-San Martin M. (2025). Development of a Novel Virus-Like Particle-Based Vaccine Against PRV-1 Suitable for DIVA Strategies. Viruses.

[102] Ganguly B., Tayshete S., Pattnaik P., Bhargav N.S.N., Kanakasapapathy A.K. (2025). A Simple Deterministic Model of Protection and Cost Benefits from Vaccinating Indian Cattle Against Infectious Bovine Rhinotracheitis. Pathogens.

[103] Gomez-Sebastian S., Perez-Filgueira D.M., Gomez-Casado E., Nunez M.C., Sanchez-Ramos I., Tabares E., Escribano J.M. (2008). DIVA diagnostic of Aujeszky’s disease using an insect-derived virus glycoprotein E. J. Virol. Methods.

[104] Xing G., Li H., Lu C., Li H., Jin Y., Yan Y., Shang S., Zhou J. (2024). The attenuated Pseudorabies virus vaccine Bartha K61 induces a weak cellular immunity: Implications for the development of PRV-vectored vaccines. Front. Immunol..

[105] Minke J.M., Fischer L., Baudu P., Guigal P.M., Sindle T., Mumford J.A., Audonnet J.C. (2006). Use of DNA and recombinant canarypox viral (ALVAC) vectors for equine herpes virus vaccination. Vet. Immunol. Immunopathol..

[106] Ayalew L.E., Kumar P., Gaba A., Makadiya N., Tikoo S.K. (2015). Bovine adenovirus-3 as a vaccine delivery vehicle. Vaccine.

[107] Wang T., Xiao Y., Yang Q., Wang Y., Sun Z., Zhang C., Yan S., Wang J., Guo L., Yan H. (2015). Construction of a gE-Deleted Pseudorabies Virus and Its Efficacy to the New-Emerging Variant PRV Challenge in the Form of Killed Vaccine. Biomed. Res. Int..

[108] Wang C.H., Yuan J., Qin H.Y., Luo Y., Cong X., Li Y., Chen J., Li S., Sun Y., Qiu H.J. (2014). A novel gE-deleted pseudorabies virus (PRV) provides rapid and complete protection from lethal challenge with the PRV variant emerging in Bartha-K61-vaccinated swine population in China. Vaccine.

[109] Yin C., Yu D., Liu T., Chen H., Yang J., Liu Z. (2025). High-yield production and preclinical protection assessment of a Chinese Hamster Ovary-K1 (CHO-K1) derived PRV gB and gD subunit vaccine against variant pseudorabies virus in pigs. Res. Vet. Sci..

[110] Ma L., Wang Y., Wang X., Zhang M., Zhu M. (2024). An isothermal recombinase polymerase assay coupled with lateral flow dipstick for differentiation of pseudorabies virus wild isolates and gE-deleted vaccine strains. Pol. J. Vet. Sci..

[111] Liu Y., Zhang S., Xu Q., Wu J., Zhai X., Li S., Wang J., Ni J., Yuan L., Song X. (2018). Investigation on pseudorabies prevalence in Chinese swine breeding farms in 2013–2016. Trop. Anim. Health Prod..

[112] Wang Y., Qiao S., Li X., Xie W., Guo J., Li Q., Liu X., Hou J., Xu Y., Wang L. (2015). Molecular epidemiology of outbreak-associated pseudorabies virus (PRV) strains in central China. Virus Genes.

[113] Wang J., Guo R., Qiao Y., Xu M., Wang Z., Liu Y., Gu Y., Liu C., Hou J. (2016). An inactivated gE-deleted pseudorabies vaccine provides complete clinical protection and reduces virus shedding against challenge by a Chinese pseudorabies variant. BMC Vet. Res..

[114] Krishnagopal A., van Drunen Littel-van den Hurk S. (2024). The biology and development of vaccines for bovine alphaherpesvirus 1. Vet. J..

[115] van Drunen Littel-van den Hurk S., Myers D., Doig P.A., Karvonen B., Habermehl M., Babiuk L.A., Jelinski M., Van Donkersgoed J., Schlesinger K., Rinehart C. (2001). Identification of a mutant bovine herpesvirus-1 (BHV-1) in post-arrival outbreaks of IBR in feedlot calves and protection with conventional vaccination. Can. J. Vet. Res..

[116] Chung Y.C., Shen H.Y., Cheng L.T., Liu S.S., Chu C.Y. (2016). Effectiveness of a BHV-1/BEFV bivalent vaccine against bovine herpesvirus type 1 infection in cattle. Res. Vet. Sci..

[117] Lee M., Reed A., Estill C., Izume S., Dong J., Jin L. (2015). Evaluation of BHV-1 antibody titer in a cattle herd against different BHV-1 strains. Vet. Microbiol..

[118] Goldkamp A.K., Kaplan B.S., Menghwar H., Kanipe C.R., Boggiatto P.M., Crawford L.S., Olsen S.C., Briggs R.E., Tatum F.M., Dassanayake R.P. (2025). Transcriptional profiles of vaccine-induced protection in bovine herpesvirus-1 and Mycoplasma bovis-challenged bison. Front. Vet. Sci..

[119] Cortese V.S., Woolums A., Thoresen M., Pinedo P.J., Short T. (2024). Mucosal immune responses in peri-parturient dairy cattle. Vet. Microbiol..

[120] Zhao C., Gao J., Wang Y., Ji L., Qin H., Hu W., Yang Y. (2022). A Novel Rabies Vaccine Based on a Recombinant Bovine Herpes Virus Type 1 Expressing Rabies Virus Glycoprotein. Front. Microbiol..

[121] Makoschey B., Beer M. (2007). A live bovine herpesvirus-1 marker vaccine is not shed after intramuscular vaccination. Berl. Munch. Tierarztl. Wochenschr..

[122] Ozkul A., Demir B., Karaoglu T., Alkan F., Dincer E., Oncel T., Burgu I. (2008). Maturation of immunoglobulin G avidity after inactive gE deleted bovine herpesvirus type 1 (BHV-1) marker vaccination. Viral Immunol..

[123] van Drunen Littel-van den Hurk S. (2006). Rationale and perspectives on the success of vaccination against bovine herpesvirus-1. Vet. Microbiol..

[124] Theurer M.E., Larson R.L., White B.J. (2015). Systematic review and meta-analysis of the effectiveness of commercially available vaccines against bovine herpesvirus, bovine viral diarrhea virus, bovine respiratory syncytial virus, and parainfluenza type 3 virus for mitigation of bovine respiratory disease complex in cattle. J. Am. Vet. Med. Assoc..

[125] Patel J.R., Heldens J. (2005). Equine herpesviruses 1 (EHV-1) and 4 (EHV-4)—Epidemiology, disease and immunoprophylaxis: A brief review. Vet. J..

[126] Patel J.R., Bateman H., Williams J., Didlick S. (2003). Derivation and characterisation of a live equid herpes virus-1 (EHV-1) vaccine to protect against abortion and respiratory disease due to EHV-1. Vet. Microbiol..

[127] Marenzoni M.L., De Waure C., Timoney P.J. (2023). Efficacy of vaccination against equine herpesvirus type 1 (EHV-1) infection: Systematic review and meta-analysis of randomised controlled challenge trials. Equine Vet. J..

[128] Stasi D., Wagner B., Barnum S., Pusterla N. (2024). Comparison of antibody and antigen response to intranasal and intramuscular EHV-1 modified-live vaccination in healthy adult horses. J. Equine Vet. Sci..

[129] Giannetto C., Giudice E., Piccione G., Castronovo C., Arfuso F. (2022). Immune and Inflammatory Response in Horse Vaccinated Against Equine Herpesviruses 1 (EHV-1) and 4 (EHV-4) Assessed by Serum Protein Electrophoretic Pattern and Leukocyte Population. J. Equine Vet. Sci..

[130] Bryant N.A., Wilkie G.S., Russell C.A., Compston L., Grafham D., Clissold L., McLay K., Medcalf L., Newton R., Davison A.J. (2018). Genetic diversity of equine herpesvirus 1 isolated from neurological, abortigenic and respiratory disease outbreaks. Transbound. Emerg. Dis..

[131] Goodman L.B., Wimer C., Dubovi E.J., Gold C., Wagner B. (2012). Immunological correlates of vaccination and infection for equine herpesvirus 1. Clin. Vaccine Immunol..

[132] Lunn D.P., Burgess B.A., Dorman D.C., Goehring L.S., Gross P., Osterrieder K., Pusterla N., Soboll Hussey G. (2024). Updated ACVIM consensus statement on equine herpesvirus-1. J. Vet. Intern. Med..

[133] Stone A.E., Brummet G.O., Carozza E.M., Kass P.H., Petersen E.P., Sykes J., Westman M.E. (2020). 2020 AAHA/AAFP Feline Vaccination Guidelines. J. Feline Med. Surg..

[134] Yoshida M., Mizukami K., Hisasue M., Imanishi I., Kurata K., Ochiai M., Itoh M., Nasukawa T., Uchiyama J., Tsujimoto H. (2022). Anaphylaxis after vaccination for cats in Japan. J. Vet. Med. Sci..

[135] Day M.J., Horzinek M.C., Schultz R.D., Squires R.A. (2016). Vaccination Guidelines Group of the World Small Animal Veterinary, A. WSAVA Guidelines for the vaccination of dogs and cats. J. Small Anim. Pract..

[136] Qi M., Yang M., Luo R., Fang L., Chen Y., Gao J., Jiao Z., Shi Y., Peng G. (2024). A novel neuro-attenuated vaccine candidate with excellent safety and protective efficacy against highly virulent Feline Herpesvirus-1. Vet. Microbiol..

[137] Tang A., Zhu M., Zhu J., Zhang D., Zhu S., Wang X., Meng C., Li C., Liu G. (2023). Pathogenicity and immunogenicity of gI/gE/TK-gene-deleted Felid herpesvirus 1 variants in cats. Virol. J..

[138] Oxman M.N., Levin M.J., Johnson G.R., Schmader K.E., Straus S.E., Gelb L.D., Arbeit R.D., Simberkoff M.S., Gershon A.A., Davis L.E. (2005). A vaccine to prevent herpes zoster and postherpetic neuralgia in older adults. N. Engl. J. Med..

[139] Cunningham A.L., Heineman T. (2017). Vaccine profile of herpes zoster (HZ/su) subunit vaccine. Expert Rev. Vaccines.

[140] Belshe R.B., Leone P.A., Bernstein D.I., Wald A., Levin M.J., Stapleton J.T., Gorfinkel I., Morrow R.L., Ewell M.G., Stokes-Riner A. (2012). Efficacy results of a trial of a herpes simplex vaccine. N. Engl. J. Med..

[141] Stanberry L.R., Spruance S.L., Cunningham A.L., Bernstein D.I., Mindel A., Sacks S., Tyring S., Aoki F.Y., Slaoui M., Denis M. (2002). Glycoprotein-D-adjuvant vaccine to prevent genital herpes. N. Engl. J. Med..

[142] Patel J.R., Heldens J.G. (2009). Immunoprophylaxis against important virus disease of horses, farm animals and birds. Vaccine.

[143] Patel J.R., Heldens J.G., Bakonyi T., Rusvai M. (2012). Important mammalian veterinary viral immunodiseases and their control. Vaccine.

[144] Eady N.A., Holmes C., Schnabel C., Babasyan S., Wagner B. (2024). Equine herpesvirus type 1 (EHV-1) replication at the upper respiratory entry site is inhibited by neutralizing EHV-1-specific IgG1 and IgG4/7 mucosal antibodies. J. Virol..

[145] Kydd J.H., Townsend H.G., Hannant D. (2006). The equine immune response to equine herpesvirus-1: The virus and its vaccines. Vet. Immunol. Immunopathol..

[146] Shi T., Ye Y., Fan Z., Yang Q., Ma Y., Zhu J. (2025). Respiratory mucosal vaccines: Applications, delivery strategies and design considerations. Biomed. Pharmacother..

[147] Wang M., Chang Q., Li B., Yang R., Chen X., Bai Y., He H., Zheng B. (2025). Engineering mucosal immunity: Advanced vaccine platforms against respiratory pathogens. Precis. Med. Eng..

[148] Wagner B., Schnabel C.L., Rollins A. (2025). Increase in Virus-Specific Mucosal Antibodies in the Upper Respiratory Tract Following Intramuscular Vaccination of Previously Exposed Horses Against Equine Herpesvirus Type-1/4. Vaccines.

[149] MacLean F., Zemek R.M., Tsegaye A.T., Graham J.B., Swarts J.L., Vick S.C., Potchen N.B., Cruz Talavera I., Warrier L., Dubrulle J. (2026). Genital herpes shedding episodes associate with altered spatial organization and activation of mucosal immune cells. JCI Insight.

[150] Aleman M., Pickles K.J., Simonek G., Madigan J.E. (2012). Latent equine herpesvirus-1 in trigeminal ganglia and equine idiopathic headshaking. J. Vet. Intern. Med..

[151] Boeren M., Meysman P., Laukens K., Ponsaerts P., Ogunjimi B., Delputte P. (2023). T cell immunity in HSV-1- and VZV-infected neural ganglia. Trends Microbiol..

[152] Treat B.R., Bidula S.M., Ramachandran S., St Leger A.J., Hendricks R.L., Kinchington P.R. (2017). Influence of an immunodominant herpes simplex virus type 1 CD8+ T cell epitope on the target hierarchy and function of subdominant CD8+ T cells. PLoS Pathog..

[153] Jones C. (2019). Bovine Herpesvirus 1 Counteracts Immune Responses and Immune-Surveillance to Enhance Pathogenesis and Virus Transmission. Front. Immunol..

[154] Lafon M. (2009). Latent viral infections of the nervous system: Role of the host immune response. Rev. Neurol..

[155] Dungu K.H.S., Malchau Carlsen E.L., Petersen O.B., Vissing N.H., Nygaard U. (2025). Herpes simplex virus—State of the art of prevention and treatment. Semin. Fetal Neonatal Med..

[156] Bergmann M., Speck S., Rieger A., Truyen U., Hartmann K. (2020). Antibody response to feline herpesvirus-1 vaccination in healthy adult cats. J. Feline Med. Surg..

[157] Jas D., Frances-Duvert V., Vernes D., Guigal P.M., Poulet H. (2015). Three-year duration of immunity for feline herpesvirus and calicivirus evaluated in a controlled vaccination-challenge laboratory trial. Vet. Microbiol..

[158] Roth J.A., Spickler A.R. (2010). Duration of immunity induced by companion animal vaccines. Anim. Health Res. Rev..

[159] Read A.F., Baigent S.J., Powers C., Kgosana L.B., Blackwell L., Smith L.P., Kennedy D.A., Walkden-Brown S.W., Nair V.K. (2015). Imperfect Vaccination Can Enhance the Transmission of Highly Virulent Pathogens. PLoS Biol..

[160] Sun Y., Zhao L., Fu Z.F. (2022). Effective cross-protection of a lyophilized live gE/gI/TK-deleted pseudorabies virus (PRV) vaccine against classical and variant PRV challenges. Vet. Microbiol..

[161] Pawaskar N.K., Kumar A., Saini M., Gupta P.K. (2026). Chitosan nanoparticle based ΔgE IBRV Vaccine: A Novel DIVA compatible strategy for enhanced immunity in guinea pigs. Int. J. Pharm..

[162] Mendoza-Morales L.F., Fiorani F., Morán K.D., Hecker Y.P., Cirone K.M., Sánchez-López E.F., Ramos-Duarte V.A., Corigliano M.G., Bilbao M.G., Clemente M. (2024). Immunogenicity, safety and dual DIVA-like character of a recombinant candidate vaccine against neosporosis in cattle. Acta Trop..

[163] Wagner B., Goodman L.B., Babasyan S., Freer H., Torsteinsdóttir S., Svansson V., Björnsdóttir S., Perkins G.A. (2015). Antibody and cellular immune responses of naïve mares to repeated vaccination with an inactivated equine herpesvirus vaccine. Vaccine.

[164] Ji C., Cai S., Lu G., Zhang G. (2020). Challenges to develop an equine herpesvirus vaccine in China. J. Infect..

[165] Spann K., Barnum S., Pusterla N. (2023). Investigation of the Systemic Antibody Response and Antigen Detection Following Intranasal Administration of Two Commercial Equine Herpesvirus-1 Vaccines to Adult Horses. J. Equine Vet. Sci..

[166] Osterrieder K., Dorman D.C., Burgess B.A., Goehring L.S., Gross P., Neinast C., Pusterla N., Hussey G.S., Lunn D.P. (2024). Vaccination for the prevention of equine herpesvirus-1 disease in domesticated horses: A systematic review and meta-analysis. J. Vet. Intern. Med..

[167] Goodman L.B., Wagner B., Flaminio M.J.B.F., Sussman K.H., Metzger S.M., Holland R., Osterrieder N. (2006). Comparison of the efficacy of inactivated combination and modified-live virus vaccines against challenge infection with neuropathogenic equine herpesvirus type 1 (EHV-1). Vaccine.

[168] Goehring L.S., Wagner B., Bigbie R., Hussey S.B., Rao S., Morley P.S., Lunn D.P. (2010). Control of EHV-1 viremia and nasal shedding by commercial vaccines. Vaccine.

[169] Wang J., Cui X., Wang X., Wang W., Gao S., Liu X., Kai Y., Chen C. (2020). Efficacy of the Bartha-K61 vaccine and a gE−/gI−/TK− prototype vaccine against variant porcine pseudorabies virus (vPRV) in piglets with sublethal challenge of vPRV. Res. Vet. Sci..

[170] Liu Y.-Y., Zhang T.-T., Zhao Y., Li N.-W., Malhi K.K., Yao X., Li J.-L. (2025). Maternal antibodies to Bartha-K61 vaccine did not protect newborn piglets from challenge with novel recombinant pseudorabies virus. Vet. J..

[171] Zhou J., Li S., Wang X., Zou M., Gao S. (2017). Bartha-k61 vaccine protects growing pigs against challenge with an emerging variant pseudorabies virus. Vaccine.

[172] Kimman T.G. (1992). Risks connected with the use of conventional and genetically engineered vaccines. Vet. Q..

[173] Endale H., Aliye S., Mathewos M. (2022). Vaccine epidemiology, evaluation, and constraints of vaccine effectiveness—A review. Vet. Vaccine.

[174] Ma B., Tao M., Li Z., Zheng Q., Wu H., Chen P. (2024). Mucosal vaccines for viral diseases: Status and prospects. Virology.

[175] Duthie M.S., Reed S.G., Kaye P.M. (2026). Chapter 00151—Immunological Properties of Adjuvants. Encyclopedia of Immunobiology.

[176] Tizard I.R., Tizard I.R. (2021). Chapter 15—Equine vaccines. Vaccines for Veterinarians.

[177] Arcidiaco S., Schreiber P., Poincelot L., Loukeri S., Lesbros C., Gueguen S. (2024). Vaccines efficacy against infection with circulating feline calicivirus after one single injection: Comparison of Leucofeligen™ FeLV/RCP and Purevax™ RCPFeLV vaccines. Vaccine.

[178] Kass P.H. (2018). Prevention of Feline Injection-Site Sarcomas: Is There a Scientific Foundation for Vaccine Recommendations at This Time?. Vet. Clin. N. Am. Small Anim. Pract..

[179] Lee J., Manon V., Chen J. (2025). Structurally diverse viral inhibitors converge on a shared mechanism to stall the antigen transporter TAP. Proc. Natl. Acad. Sci. USA.

[180] Chai H.H., Kim T.H., Kim Y.R., Lim D. (2021). Structure and function of the porcine TAP protein and its inhibition by the viral immune evasion protein ICP47. Int. J. Biol. Macromol..

[181] Wachalska M., Riepe C., Slusarz M.J., Graul M., Borowski L.S., Qiao W., Foltynska M., Carette J.E., Bienkowska-Szewczyk K., Szczesny R.J. (2024). The herpesvirus UL49.5 protein hijacks a cellular C-degron pathway to drive TAP transporter degradation. Proc. Natl. Acad. Sci. USA.

[182] Karska N., Zhukov I., Lipinska A.D., Rodziewicz-Motowidlo S., Krupa P. (2023). Why does the herpes simplex 1 virus-encoded UL49.5 protein fail to inhibit the TAP-dependent antigen presentation?. Biochim. Biophys. Acta Biomembr..

[183] Foight G.W., Keating A.E. (2015). Locating Herpesvirus Bcl-2 Homologs in the Specificity Landscape of Anti-Apoptotic Bcl-2 Proteins. J. Mol. Biol..

[184] Bryant N.A., Davis-Poynter N., Vanderplasschen A., Alcami A. (2003). Glycoprotein G isoforms from some alphaherpesviruses function as broad-spectrum chemokine binding proteins. EMBO J..

[185] Alcami A. (2003). Viral mimicry of cytokines, chemokines and their receptors. Nat. Rev. Immunol..

[186] Awasthi S., Lubinski J.M., Friedman H.M. (2009). Immunization with HSV-1 glycoprotein C prevents immune evasion from complement and enhances the efficacy of an HSV-1 glycoprotein D subunit vaccine. Vaccine.

[187] Komala Sari T., Gianopulos K.A., Nicola A.V. (2020). Glycoprotein C of Herpes Simplex Virus 1 Shields Glycoprotein B from Antibody Neutralization. J. Virol..

[188] Chowdhury S.I. (1997). Fine mapping of bovine herpesvirus 1 (BHV-1) glycoprotein C neutralizing epitopes by type-specific monoclonal antibodies and synthetic peptides. Vet. Microbiol..

[189] Huemer H.P., Nowotny N., Crabb B.S., Meyer H., Hubert P.H. (1995). gp13 (EHV-gC): A complement receptor induced by equine herpesviruses. Virus Res..

[190] Huemer H.P., Strobl B., Nowotny N. (2000). Use of apathogenic vaccinia virus MVA expressing EHV-1 gC as basis of a combined recombinant MVA/DNA vaccination scheme. Vaccine.

[191] Zeng L. (2016). Mucosal adjuvants: Opportunities and challenges. Hum. Vaccin. Immunother..

[192] Lavelle E.C., Ward R.W. (2022). Mucosal vaccines—Fortifying the frontiers. Nat. Rev. Immunol..

[193] Kweon M.N. (2014). Recent progress in mucosal immunology and vaccine development. Exp. Mol. Med..

[194] Raychaudhuri S.P., Raychaudhuri S.K. (2026). Is psoriatic arthritis a dissemination of cutaneous psoriatic TRM cells? Role of tissue-resident memory T cells (TRM) in psoriatic disease. Curr. Opin. Immunol..

[195] Hubaishi F., Karkout R., Labrie L., Alyazidi R.M., Solomon M., Aldossary H., Ward B.J., Fixman E.D. (2026). Epicutaneous application of house dust mite induces allergic skin inflammation and atopic march to the lung upon airway allergen challenge. Clin. Immunol..

[196] Mackay L.K., Rahimpour A., Ma J.Z., Collins N., Stock A.T., Hafon M.L., Vega-Ramos J., Lauzurica P., Mueller S.N., Stefanovic T. (2013). The developmental pathway for CD103(+)CD8+ tissue-resident memory T cells of skin. Nat. Immunol..

[197] Gebhardt T., Wakim L.M., Eidsmo L., Reading P.C., Heath W.R., Carbone F.R. (2009). Memory T cells in nonlymphoid tissue that provide enhanced local immunity during infection with herpes simplex virus. Nat. Immunol..

[198] Carneiro F.A., Cortines J.d.R., Essus V.A., da Silva I.B.N., Prajapati V. (2022). Chapter 4—Vaccine engineering & structural vaccinology. System Vaccinology.

[199] Samal S.K., Das J., Dave S., Soares S.d.C., Tiwari S. (2024). Chapter 12—Structural vaccinology approaches to enhance efficacy, stability, and delivery of protective antigens. Reverse Vaccinology.

[200] Unger P.A., Oja A.E., Khemai-Mehraban T., Ouwendijk W.J.D., Hombrink P., Verjans G. (2022). T-cells in human trigeminal ganglia express canonical tissue-resident memory T-cell markers. J. Neuroinflamm..

[201] Han Q., Dong J., Ren W., Qu Y., Wei F. (2026). Preparation and evaluation of multi-functional Nimbolide-loaded PLGA/chitosan nanoparticles as a potential therapy for renal cell carcinoma through activation of intrinsic apoptotic pathway. Int. J. Biol. Macromol..

[202] Al-Halifa S., Gauthier L., Arpin D., Bourgault S., Archambault D. (2019). Nanoparticle-Based Vaccines Against Respiratory Viruses. Front. Immunol..

[203] Liu S., Hu M., Liu X., Liu X., Chen T., Zhu Y., Liang T., Xiao S., Li P., Ma X. (2023). Nanoparticles and Antiviral Vaccines. Vaccines.

[204] Cohen J.I. (2024). Therapeutic vaccines for herpesviruses. J. Clin. Investig..

[205] Hernández M., Caltempa A.O., Villalobos N., Elisondo M.C., Guzmán C., Hernández-Aceves J.A., Aguilar L., Flores-Pérez I., Martínez J.J., Elguea Zarate S.D. (2025). Novel Oral papaya-based preventive and therapeutic vaccine for controlling different zoonotic Taeniid parasites: Current advances. Vaccine.

