# Supplementary material for: A Comparative Review of Veterinary and Human Vaccine Development Strategies: Insights into Herpesvirus Vaccinology from Latency to Elimination
Source: Vaccines (Basel). 2026 Mar 7;14(3):249. doi: 10.3390/vaccines14030249 (PMC13030153; doi:10.3390/vaccines14030249)
Supplement: Supplementary file 1 [file vaccines-14-00249-s001.zip › vaccines-4163404-supplementary.pdf]

Table S1. **Comparative Frameworks in Human and Veterinary Vaccinology of Alphaherpesvirus**[a]

Feature	HSV/VZV	PRV	BHV-1	EHV-1/4	FHV-1
Primary Objective	Individual protection; Quality of Life (QoL); Prevention of chronic pain/oncogenesis.	Herd-wide eradication; facilitation of international trade.	Reducing morbidity in feedlots (shipping fever); maintaining milk/meat yield.	Protecting high-value individuals; preventing "abortion storms" in breeding.	Reducing clinical signs in domestic cats; shelter population control.
Acceptable Risk Profile	**Extremely Low:** Even mild transient side effects (reactogenicity) can stall a Phase III trial.	**Moderate:** Minor growth delays or transient fever are acceptable if herd protection is high.	**Context-Dependent:** Some LAV-induced abortion risk is tolerated in high-prevalence non-breeding herds.	**Low-Moderate:** High-value athletes require high safety; injection site soreness is a concern.	**Specific Risk:** High sensitivity to adjuvants; concerns over Feline Injection-Site Sarcoma (FISS).
Regulatory Framework[b]	Stringent (FDA/EMA); requires massive, multi-year, multi-phase clinical trials.	Regulatory-driven; focuses on DIVA compliance and herd-level shedding reduction.	Focus on efficacy against field challenge and "marker" gene stability for trade.	National equestrian federations often mandate vaccination for competition entry.	Core vaccine recommendations by veterinary associations (WSAVA).
Feasibility of Eradication	**Low:** Ubiquitous social transmission; ethical impossibility of "culling" or total isolation.	**High:** Demonstrated success via DIVA-LAV and strict movement controls.	**Moderate:** Feasible in closed regional herds; difficult in global, open-market cattle trade.	**Low:** Frequent movement and performance stress lead to constant subclinical cycling.	**Negligible**: High percentage of subclinical carriers in the global domestic population.
Safety Thresholds	Zero tolerance for reversion to virulence or integration into the host genome.	Focus on preventing recombination with wild-type field strains.	Focus on safety for pregnant animals and preventing systemic spread to the CNS.	Prevention of vaccine-induced vasculitis or neurological complications.	Minimizing chronic inflammation at the site of subcutaneous injection.
Immunological CoP	$CD4^+$ T-cell frequency (VZV); $CD8^+T_{RM}$ (HSV-2).	Neutralizing antibodies and gE-negative seroconversion.	Combined IgG/IgA (nasal) and robust $CD8^+$ T-cell surveillance.	High neutralizing IgG (abortion) and $CD8^+$ CTLs	Mucosal IgA and reduction of Th2-driven inflammation.

a: **Comparative Analysis**

While the molecular architecture of the *Alphaherpesvirinae* is remarkably conserved, the social, economic, and regulatory environments in which their vaccines operate are vastly different. Understanding these divergences is critical for the ‘One Health’ approach. The dichotomy between human and veterinary vaccine development is most evident in the Safety-to-Efficacy Ratio. In swine and bovine medicine, the "herd-as-an-organism" philosophy prevails. A vaccine that causes minor weight loss in 2% of piglets but prevents a 50% mortality outbreak is a clear economic winner. In contrast, a human HSV-2 vaccine that causes even a 0.1% of significant adverse events would likely fail to receive regulatory approval, even if it provided 70% protection. For PRV and BHV-1, a vaccine without a marker gene is often legally and economically ‘useless’ in regions pursuing eradication, regardless of its immunological potency. In human medicine, DIVA is currently a secondary concern, as the primary goal is the relief of individual suffering rather than the certification of a "pathogen-free" population for trade purposes. As we move toward mRNA and VLP technologies (discussed in Section 7). While mRNA allows for the rapid multiplexing of antigens (targeting gD, gB, and gH simultaneously), the cost of certifying such a complex ‘cocktail’ under human FDA guidelines is exponentially higher than the USDA requirements for a veterinary equivalent. Conversely, the eradication feasibility continues to serve as the guiding flag for livestock herpesvirus control efforts. Because animals can be moved, tested, and—if necessary—culled, the veterinary sector acts as the primary testing ground for "leaky" vaccines. The lessons learned from the emergence of EHV-1 variants under vaccine pressure provide a sobering warning for human HSV-2 research: any vaccine that reduces symptoms without blocking transmission may inadvertently drive the evolution of more virulent or neurotropic strains.

b: The regulatory authorities worldwide include USDA-CVB (U.S.A), EMA-CMVP (European Union), WOAH (international organization, formerly OIE) and VICH (USA, EU and Japan plus observers from other nations or regions).
